# Obligatory Exercise and Eating Attitudes—A Pilot Study on Polish Adolescents

**DOI:** 10.3390/jcm15145455

**Published:** 2026-07-12

**Authors:** Magdalena Jaroch-Lidzbarska, Konrad Hryniewicz, Piotr Sawicki, Dominika Wilczyńska

**Affiliations:** 1 Department of Physical Culture, Gdansk University of Physical Education and Sport, Kazimierza Górskiego 1, 80-336 Gdansk, Poland; piotr.sawicki@awf.gda.pl; 2 Department of Marketing and Quantitative Methods, Faculty of Management and Quality Science, Gdynia Maritime University, Morska 81/87, 81-225 Gdynia, Poland; metodolog.pl@gmail.com; 3 Faculty of Social and Humanities, WSB Merito University, Grunwaldzka 238A, 80-266 Gdansk, Poland; dominika.wilczynska@gdansk.merito.pl

**Keywords:** obligatory exercise, adolescents, athletes, psychiatric patients, eating disorders

## Abstract

**Background:** This pilot study examined differences in levels of obligatory exercise between two groups of adolescents: psychiatric patients diagnosed with eating disorders and youth engaged in qualified sports. **Methods:** The study included 45 adolescent psychiatric patients with eating disorders and 45 students training in sports departments (track and field, judo, gymnastics), aged 14–20 years. The sample comprised 50 women, 28 men, 10 non-binary participants, and two individuals who declined to identify their gender. The following instruments were used: the Eating Attitudes Test (EAT-26), Exercise Dependence Questionnaire (EDQ), Obligatory Exercise Questionnaire (OEQ), and Inventory of Physical Activity Objectives (IPAO). Between-group differences were analyzed using the Mann–Whitney U test. Most scales demonstrated acceptable internal consistency (Cronbach’s α > 0.70), although several scales showed lower reliability estimates. **Results:** Adolescents diagnosed with eating disorders showed significantly higher levels of problematic eating behaviours (rg = 0.94), exercise fixation (rg = 0.71), exercise commitment (rg = 0.33), withdrawal symptoms (rg = 0.52), exercise for weight control (rg = 0.83), and insight into problems (rg = 0.77) than athletes. In contrast, athletes reported higher levels of exercise for social (rg = 0.74) and health-related reasons (rg = 0.40). Effect sizes ranged from moderate to very large. These findings indicate marked differences in the psychological motives and compulsive characteristics of exercise between adolescents with eating disorders and athletes. **Conclusions:** This pilot study suggests that adolescents with eating disorders differ from athletes primarily in the psychological characteristics and motives underlying exercise rather than in exercise volume alone. These findings may help inform the assessment of problematic exercise in clinical practice, although they require confirmation in larger, adequately controlled studies.

## 1. Introduction

Involvement in physical activity offers numerous benefits for both physical and mental health. However, excessive exercise can have negative consequences that affect daily life and potentially lead to behavioural addiction. Exercise dependence, as first defined by de Coverley Veale [1], refers to physical activity with a regular schedule—often one or more times daily—where the individual increasingly prioritizes exercise over other activities. According to this definition, exercise-dependent individuals may experience withdrawal symptoms, such as mood disturbances, upon stopping their exercise routine, but they find relief from these symptoms with further exercise. Recent evidence suggests that exercise can become obsessive, compulsive, or addictive when individuals begin to experience negative physiological and psychological symptoms, including withdrawal symptoms, exercising despite injury, and impaired social functioning. Research indicates a developmental pathway in which exercise evolves from an enjoyable activity (‘want to’) to a more obligatory commitment (‘have to’) and, ultimately, to a psychophysiological dependence (‘must do’) that is no longer tied to the pleasure derived from physical activity [2]. Various terms are used to describe this phenomenon, such as exercise addiction, exercise dependence, compulsive exercise, and obligatory or abusive exercise. The most commonly used term is exercise addiction (EA) [3].

According to Griffiths (adapted from Brown) [4], exercise addiction can be conceptualized as a behavioural addiction characterized by six core components: salience, tolerance, mood modification, withdrawal, conflict, and relapse. Together, these components describe a pattern in which exercise becomes the dominant activity in an individual’s life, requires increasing engagement to achieve the desired effects, serves as a means of regulating mood, produces psychological or physical discomfort when reduced or discontinued, generates interpersonal or intrapsychic conflicts, and is characterized by a tendency to return to excessive exercise following periods of control or abstinence.

In the present study, Griffiths’ model serves as the theoretical framework for understanding obligatory exercise as a potentially addictive behaviour. However, the instruments used—the Exercise Dependence Questionnaire (EDQ) and the Obligatory Exercise Questionnaire (OEQ)—were not designed to operationalize the six components of this model directly. Instead, they assess complementary cognitive, emotional, and behavioural aspects of exercise dependence and obligatory exercise, including exercise commitment, interference with social functioning, withdrawal symptoms, exercise for weight control, and problematic eating behaviours.

EA can exist as a primary (driven by motivation associated with dedicated exercise) or secondary (with the common simultaneous co-occurrence of eating disorders) dependence problem [1]. In primary dependence, exercising is frequent and intense in anticipation of solving a significant problem or escaping from stress. In secondary exercise dependence, compensatory behaviour occurs, and exercising cannot be controlled to achieve the desired physical appearance, control, and weight loss. Furthermore, it is treated as a tool necessary to achieve the abovementioned goals [5]. Elbourne and Chen (2007) demonstrated the co-occurrence of both disorders in the continuum model of obligatory exercise, which suggests that a significant preoccupation with food and concern over weight and shape, which results in eating disorders, may be the extreme end of obsessive attitudes towards exercise [6]. Although this distinction provides a useful theoretical framework, the present study was not designed to classify participants as having primary or secondary exercise dependence.

Several studies have reported the co-occurrence of obligatory exercise with other mental health disorders, specifically, not only eating disorders but also other behavioural and chemical addictions [7].

Adolescents with eating disorders and adolescent athletes constitute two populations in which high levels of physical activity are frequently observed; however, the psychological meaning of exercise may differ substantially between these groups. In athletes, intensive exercise is often related to training demands, performance goals, and social participation, whereas in adolescents with eating disorders excessive exercise may serve compensatory functions related to weight control and eating disorder psychopathology. Comparing these groups may therefore help distinguish exercise behaviours associated with sport participation from those reflecting psychological mechanisms involved in eating disorders. Such differentiation may be clinically relevant because compulsive exercise has been identified as a potential factor contributing to the maintenance of eating disorder symptoms and poorer treatment outcomes. Furthermore, data on obligatory exercise among Polish adolescents remain scarce. Therefore, the aim of the present study was to compare adolescents diagnosed with eating disorders and adolescents engaged in qualified sports with regard to obligatory exercise, exercise-related attitudes and behaviours, and eating attitudes. Based on previous research, we hypothesized that adolescents with eating disorders would report (1) higher levels of obligatory exercise and exercise-related psychological characteristics associated with compulsive exercise (e.g., exercise fixation, withdrawal symptoms, and exercise for weight control) and (2) higher levels of problematic eating attitudes than adolescents engaged in qualified sports.

## 2. Methods

### 2.1. Participants and Selection Criteria

A total of 45 adolescents diagnosed with eating disorders and 45 athletes engaging in judo, gymnastics and track and field were recruited for this study between 1 February 2024 and 31 May 2024. The final sample consisted of 90 participants recruited from these two research groups. The first group consisted of in-patients from the psychiatric ward for children and adolescents of the Voivodeship Psychiatric Hospital in Gdańsk. The second group comprised adolescent athletes from the Academic Sports Club of Gdańsk University of Physical Education and Sport. The young athletes have been trained in judo, track and field and gymnastics. The clinical group comprised adolescent psychiatric in-patients with a current diagnosis of an eating disorder established according to ICD-10 diagnostic criteria. The group included patients diagnosed with anorexia nervosa, bulimia nervosa, binge eating disorder, and unspecified eating disorders. Detailed information regarding the distribution of diagnoses, illness stage, treatment phase, and medication use was not available. Participants were asked to report their gender (woman, man, non-binary, or preferred not to answer). The sample consisted of male (31%), female (56%) and non-binary (11%) participants, and two of the research participants refused to identify their gender (2%). The mean age of the entire research group was *M* = 16.62 years (*SD* = 2.05). The average stature of the athletes was 170.8 cm, and the average stature for the group of adolescents with eating disorders was 163.67 cm. The average body mass of the athletes and the patients was 60.76 kg and 50.6 kg, respectively. Among the adolescent athletes, 67% spent more than 8 h per week on training, 22% trained from 6 to 8 h, 8.9% trained from 4 to 6 h, and 2.2% were involved in physical activity for only 1–2 h per week. In contrast, only 4.4% of the psychiatric patients exercised for more than 8 h per week, 6.7% exercised for 6–8 h, 36% engaged in physical activity from 4 to 6 h, 38% exercised for 2–4 h, and 16% were active for 1–2 h per week. The inclusion criteria for the group of adolescents with eating disorders were an age between 14 and 20 years, having a current psychiatric diagnosis of an eating disorder, not practising qualified sports and providing informed consent for participation in the research or the consent of a parent/legal guardian in the case of someone underage. The patients were excluded if they did not meet the age (14–20 years) criterion, practised qualified sports, did not have a psychiatric diagnosis of an eating disorder, did not give informed consent to participate in the research, their parent/legal guardian did not agree to participate in the case of someone underage, or they withdrew their consent. In the research group of adolescent athletes, the criteria for participants included respondents who practised qualified sports, were aged 14–20 years, and provided informed consent for their participation in the research, or in the case of someone underage, a parent/legal guardian provided consent. The meaningful criterion was lack of a diagnosis of eating disorders, which we determined by a question at the beginning of our survey: ‘Have you ever been diagnosed with an eating disorder by a physician?’ The adolescents who did not meet the age criterion, were not involved in practising qualified sports, did not consent to participate in the research, withdrew their consent, whose parent/legal guardian did not agree to participation in the case of someone underage, or were diagnosed with any type of eating disorders were excluded from the research. Prior to the study, all participants provided written informed consent or the parents/guardians of the underage participants provided informed consent. The study was conducted with the approval of the Bioethical Committee of Gdansk University of Physical Education and Sport (No. 2/2024) and in accordance with the Declaration of Helsinki. All of the participants were informed that they could withdraw at any time from the research survey without any repercussions.

### 2.2. Study Design

The research survey was conducted using an online questionnaire, which took approximately 15–20 min and was administered in the presence of a researcher. The online survey was completed by the participants individually. Each responder’s motivation to complete the survey was supported by the presence of the researcher and the information that after finishing, they would be rewarded with a small gift (a colourful sticker to choose from for the adolescents with eating disorders and protein bars and sweets for the athletes).

### 2.3. Measurements

The research was an observational study and was conducted via a quantitative method with an author’s survey, which consisted of the following instruments:

1. The Polish version of the Eating Attitudes Test (EAT-26) (Rogoza, Brytek-Matera & Garner, 2016) [8] was used to assess the eating behaviours of the study participants. The test consists of three subscales concerning Dieting (e.g., ‘Aware of the calorie content of the foods that I eat’), Bulimia and Food Preoccupation (e.g., ‘Have gone on eating binges where I feel that I may not be able to stop’), and Oral Control (e.g., ‘Avoid eating when I am hungry’). The participants responded to 26 items on a six-point Likert scale (1—never; 6—always). Responses were recoded according to the standard EAT-26 scoring procedure so that higher scores reflected greater eating disorder symptomatology. For statistical analyses, the mean score across all items was calculated, with higher scores indicating more problematic eating attitudes and behaviours.
2. The Exercise Dependence Questionnaire (EDQ) (Ogden, Veale & Summers, 1997) [9], which was translated into Polish with independent forward-translation by two translators (both health care professionals) and reconciliation but without back translation [10], was used to assess the motivation to continue exercising. The test contains the following eight subscales: interference with social/family life (e.g., ‘My level of exercising makes me tired at work’), positive reward (e.g., ‘After an exercise session, I feel more positive about myself’), withdrawal symptoms (e.g., ‘If I cannot exercise, I feel agitated’), exercise for weight control (e.g., ‘I exercise to control my weight’), insight into problems (e.g., ‘My exercising is ruining my life’), exercise for social reasons (e.g., ‘I exercise to meet other people’), exercise for health reasons (e.g., ‘I exercise to feel fit’) and stereotyped behaviour (e.g., ‘My weekly pattern of exercise is repetitive’). The participants responded to 29 items on a seven-point Likert scale (1—strongly disagree; 7—strongly agree).
3. The Obligatory Exercise Questionnaire (OEQ) (Pasman & Thompson, 1988) [11] was used to measure the respondents’ attitudes related to exercising. The translation of the test into Polish was conducted in the same manner as the procedure for the previous test. The Obligatory Exercise Questionnaire has previously been used in a Polish–Chinese comparative study of adults, where it demonstrated good internal consistency (Cronbach’s α = 0.854) [12]. However, this estimate was obtained in an adult sample and may not be directly generalizable to adolescents. The test contains three subscales as follows: exercise fixation (e.g., ‘When I don’t exercise, I feel guilty’), exercise frequency (e.g., ‘I exercise more than three days per week’) and exercise commitment (e.g., ‘When I miss an exercise session, I feel concerned about my body possibly getting out of shape’). The questionnaire includes 20 items (e.g., ‘I frequently push myself to the limit’). The participants marked how often they experienced each physically active situation on a four-point Likert scale (1—never; 4—always). Those who achieved higher scores on the questionnaire indicated a more relevant obligation to exercise [13].
4. Two items derived from the Inventory of Physical Activity Objectives (IPAO) (Lipowski & Zaleski, 2015) [14] were used to obtain descriptive information regarding participants’ physical activity (exercise duration and participation in organized classes): (1) ‘Do you participate in classes (e.g., in a fitness club/gym)?’—yes/no response; if yes, ‘How many times per month?’—open question; and (2) ‘How long, do you regularly (without longer breaks) engage in physical activity?’—open question. The full IPAO was not administered because these variables served only to characterize the sample and were not analyzed as IPAO scale scores.

### 2.4. Statistical Analysis

Data analysis was performed via SZTOS (Version SZTOSik.1) statistical software (Hryniewicz, Milewska, 2023) [15]. The software is written in R and serves solely to report, visualize, and tabulate the results of statistical analyses. The analytical results obtained with SZTOS are consistent with those generated using corresponding packages and functions available in R. The study sample characteristics in Table 1 consisted of 90 participants aged 14–20 years, divided into two equal groups: athletes and adolescents with eating disorders (ED). Overall, the participants were mostly students, with the largest proportion attending high school, and the majority reported not being employed. The two groups were comparable in terms of age, employment status, and highest lifetime body weight. Significant between-group differences were observed in sex distribution, type of school, weekly physical activity, participation in organized training, competition participation, ED diagnosis, lowest lifetime weight, height, current weight, ideal weight, and BMI. Athletes were more often male, reported higher levels of weekly physical activity, more frequently participated in organized training, and all reported taking part in competitions. In contrast, adolescents with ED were more often female or non-binary, reported substantially lower levels of physical activity, less frequent participation in organized training, and no participation in competitions. By definition, all participants in this group had received a medical diagnosis of an eating disorder. The adolescents with ED were mainly characterized by a predominance of female participants, a higher proportion of non-binary individuals compared with athletes, a confirmed eating disorder diagnosis, lower current body weight, lower lowest lifetime weight, and lower BMI. They were also shorter on average and reported lower weekly physical activity than athletes. These findings indicate that the adolescents with ED group differed from the athletes group primarily in clinical status, sex distribution, body-weight-related characteristics, and physical activity patterns.

## 3. Results

**Figure 1:**
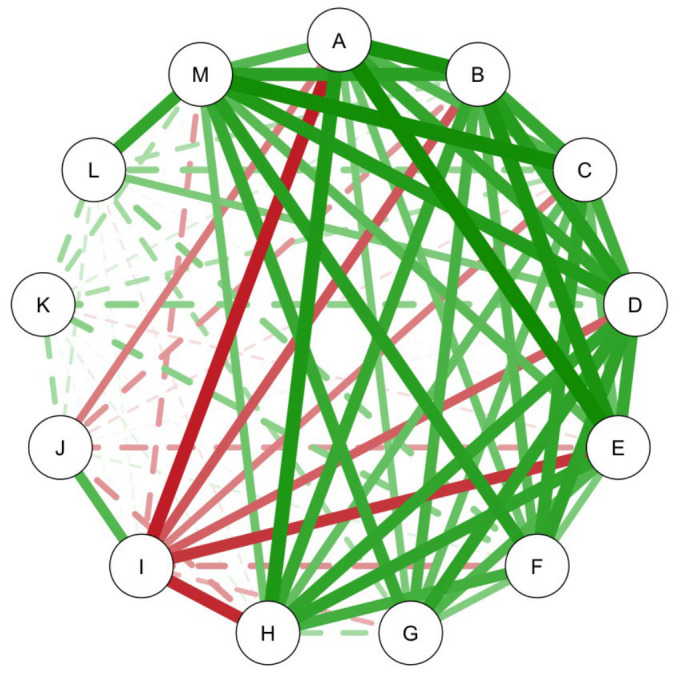
A visualization of the relationships among the variables of problematic eating behaviours (EAT-26), the Exercise Dependence Questionnaire (EDQ) subscales and the Obligatory Exercise Questionnaire (OEQ). Note: A = EAT, B = exercise fixation, C = exercise commitment, D = withdrawal symptoms, E = exercising for weight control, F = interference with social life, G = positive reward, H = insight into problem, I = exercise for social reasons, J = exercise for health reasons, K = stereotyped behaviour, L = exercise frequency, M = Obligatory Exercise Questionnaire. The darker the green colour = the more positive the correlation; the darker the red colour = the more negative the correlation. The dashed line indicates a nonsignificant relationship between variables. The solid line indicates a significant relationship between variables. The figure is based on the obtained estimates of Spearman correlation coefficients.

**Figure 2:**
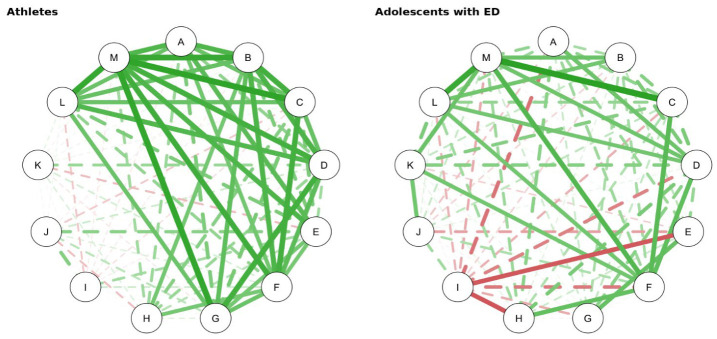
A visualization of the relationships among the variables between groups. Note: A = EAT, B = exercise fixation, C = exercise commitment, D = withdrawal symptoms, E = exercising for weight control, F = interference with social life, G = positive reward, H = insight into problem, I = exercise for social reasons, J = exercise for health reasons, K = stereotyped behaviour, L = exercise frequency, M = Obligatory Exercise Questionnaire. The darker the green colour = the more positive the correlation; the darker the red colour = the more negative the correlation. The dashed line indicates a nonsignificant relationship between variables. The solid line indicates a significant relationship between variables. The figure is based on the obtained estimates of Spearman correlation coefficients.

To evaluate the internal consistency of the study instruments, Cronbach’s alpha and McDonald’s omega coefficients were calculated (Table 2). Overall, most scales demonstrated acceptable to excellent internal consistency. The highest reliability was observed for the EAT-26, withdrawal symptoms, exercise for weight control, and insight into problems scales. Lower reliability was found for exercise commitment, exercise frequency, and exercise for social reasons; therefore, findings based on these scales should be interpreted with caution. Detailed reliability analyses are presented in Figures S1–S13. The distributions of the analyzed variables were examined prior to statistical analyses. Because the assumption of normality was not consistently met (Tables S2–S4, Figures S14–S26), non-parametric methods were applied throughout the study. Spearman rank-order correlations were calculated to examine associations between eating pathology and exercise-related variables (Table 3). To account for multiple testing, Holm’s correction was applied. Overall, eating pathology (EAT-26) was most strongly associated with exercise for weight control (ρ = 0.82, *p* < 0.001), exercise fixation (ρ = 0.77, *p* < 0.001), and insight into problems (ρ = 0.73, *p* < 0.001). Moderate positive correlations were observed with withdrawal symptoms (ρ = 0.63), exercise commitment (ρ = 0.51), and the total Obligatory Exercise Questionnaire score (ρ = 0.50). The overall pattern of correlations is illustrated in Figure 1. In contrast, exercise frequency and stereotyped behaviour showed little or no association with EAT-26 scores. Exercise for social reasons and exercise for health reasons were negatively associated with eating pathology. The total OEQ score was most strongly associated with exercise commitment (ρ = 0.80) and withdrawal symptoms (ρ = 0.71). Correlation coefficients were additionally compared between adolescents with eating disorders and athletes. After Holm correction for multiple comparisons, only two between-group differences remained statistically significant. These between-group differences are illustrated in Figure 2. Exercise for weight control was negatively associated with exercise for social reasons among adolescents with eating disorders but not among athletes. In contrast, positive reward was strongly associated with obligatory exercise among athletes but not among adolescents with eating disorders. The complete comparison of correlation coefficients is presented in Supplementary Table S1. Group differences between adolescents with eating disorders and athletes are presented in Table 4. Mann–Whitney U tests showed that adolescents with eating disorders scored significantly higher on problematic eating behaviours (EAT-26), exercise fixation, withdrawal symptoms, exercise commitment, exercise for weight control, and insight into problems. Athletes scored significantly higher on exercise for social reasons and exercise for health reasons. Following Holm correction for multiple comparisons, the differences in exercise frequency, interference with social life, and the total Obligatory Exercise Questionnaire score were no longer statistically significant. Exercise commitment remained statistically significant after correction (adjusted *p* = 0.039), although this finding should be interpreted with caution because of the relatively low internal consistency of this scale.

To determine the between-group differences in the thirteen outcome variables, they were examined with Mann–Whitney U tests, as the assumptions of parametric tests were violated (Supplementary Materials presents detailed diagnostic plots, variance and normality). To control the *p*-values within this series of comparisons, they were adjusted using, as previously, the Holm procedure. All *p*-values reported in the text, tables, and figures are Holm-adjusted. Effect sizes were quantified with the rank-biserial correlation, computed as rg = 1 − 2 U/(n_1_n_2_) [16], and interpreted according to conventions for correlation-type effect sizes, with |rg| < 0.10 considered negligible, 0.10–0.29 small, 0.30–0.49 moderate, and ≥0.50 large [17]. The results of the Mann–Whitney U tests, together with descriptive statistics and rank-biserial correlation effect sizes, are presented in Table 4. Adolescents with eating disorders scored significantly higher than athletes on the EAT-26, U = 60.00, *p* < 0.001, r = 0.94 (large effect); exercising for weight control, U = 167.50, *p* < 0.001, r = 0.83 (large); insight into the problem, U = 229.50, *p* < 0.001, r = 0.77 (large); exercise fixation, U = 290.50, *p* < 0.001, r = 0.71 (large); withdrawal symptoms, U = 486.50, *p* < 0.001, r = 0.52 (large); and exercise commitment, U = 679.50, adjusted *p* = 0.039, r = 0.33 (moderate). In contrast, athletes scored significantly higher on exercising for social reasons, U = 263.00, *p* < 0.001, r = 0.74 (large), and exercising for health reasons, U = 608.00, adjusted *p* = 0.008, r = 0.40 (moderate). Three comparisons that were nominally significant without correction did not remain so after Holm adjustment: exercise frequency, U = 719.50, *p* = 0.079, rg = 0.29 (small); interference with social life, U = 750.50, *p* = 0.138, rg = 0.26 (small); and the Obligatory Exercise Questionnaire total score, U = 756.00, *p* = 0.138, rg = 0.25 (small). Given their small effect sizes and adjusted *p*-values, these differences should be regarded as inconclusive rather than as evidence of group equivalence. The remaining comparisons were nonsignificant, with small-to-negligible effects (positive reward: U = 802.50, *p* = 0.180, rg = 0.21; stereotyped behaviour: U = 944.00, *p* = 0.575, rg = 0.07). The difference in exercise commitment remained statistically significant after Holm correction; however, because the adjusted *p*-value was close to the significance threshold, this finding should be interpreted with caution. Taken together, the groups differed most markedly in exercise motives (weight control versus social and health reasons) and in the compulsive features of exercise. In contrast, no reliable between-group differences remained for overall exercise involvement, exercise frequency, or the total OEQ score after Holm correction for multiple comparisons.

## 4. Discussion

The aim of the study was to compare adolescents with eating disorders and adolescent athletes with regard to obligatory exercise, exercise-related attitudes and behaviours, and eating attitudes. The findings of this study indicate significant differences between adolescents with eating disorders and adolescents engaged in qualified sports across several dimensions of obligatory exercise, exercise-related attitudes and behaviours, and eating attitudes.

These findings should be interpreted in light of the study design. The comparison was conducted between two distinct populations—adolescents diagnosed with eating disorders who did not participate in qualified sports, and adolescents engaged in qualified sports without an eating disorder diagnosis. Consequently, eating disorder status and athletic status were not independent, and the observed between-group differences cannot be attributed solely to eating disorder status. Rather, they reflect differences between these two populations.

The higher levels of problematic eating behaviours and exercise for weight control observed among adolescents with eating disorders are consistent with the clinical characteristics of eating disorders. However, given the study design, these differences should not be interpreted as reflecting the independent effect of eating disorder status. Lichtenstein et al. (2018) [18] found that 21% of youth patients with eating disorders reported obligatory exercise. This finding indicates a notable co-occurrence of disordered eating and compulsive exercise behaviours, particularly among adolescents with existing eating disorder diagnoses. An older study by Blaydon and Lindner (2002) [19] that compared adolescents involved in sports and adolescents with eating disorders suggested that both groups may engage in problematic exercise behaviours, but athletes often experience obligatory exercise as part of a performance-driven context, which is distinct from the pathology of individuals primarily diagnosed with eating disorders. Moreover, a systematic review by Godoy-Izquierdo et al. (2021) [20] highlighted that obligatory exercise is common in the context of disordered eating among athletes, supporting the view that excessive exercise can be integral to the pathology of eating disorders.

In contrast, the elevated scores on the subscales for withdrawal symptoms, exercise fixation, and insight into problems observed in the group of adolescents with eating disorders compared with athletes may be attributed to more severe personality traits such as compulsiveness, perfectionism, and rigidity. For example, Hauck et al. (2020) [21] investigated amateur endurance athletes and identified links between exercise dependence and disordered eating patterns, which highlights exercise dependence as a mediator influenced by perfectionism. Higher levels of insight into problems may reflect greater awareness of the negative consequences associated with compulsive exercise and eating disorder psychopathology.

From the perspective of eating disorder psychopathology, the present findings may suggest that obligatory exercise is not merely a behavioural manifestation of increased physical activity but may reflect broader psychological processes. The previous literature has linked compulsive exercise in individuals with eating disorders to perfectionism, cognitive rigidity, compulsivity, body image concerns, and difficulties in emotion regulation. Although these constructs were not directly assessed in the present study, the higher levels of exercise fixation, withdrawal symptoms, insight into problems, and exercise for weight control observed among adolescents with eating disorders may be consistent with the hypothesis that excessive exercise serves important psychological functions within the eating disorder process.

The high correlations observed between the EAT-26 and selected exercise-related measures in the pooled sample should also be interpreted with caution. Because the study compared two markedly different populations, these coefficients partly reflect between-group differences in addition to within-group associations. Indeed, the corresponding correlations were substantially lower when calculated separately for adolescents with eating disorders and athletes, suggesting that the instruments assess related but distinct constructs rather than measuring the same underlying phenomenon. Thus, the observed overlap appears to reflect shared psychological features of eating pathology and obligatory exercise rather than redundancy of the measures.

One possible interpretation is that exercise may function as a maladaptive strategy for regulating distress and maintaining a sense of control over body weight and shape. In this context, obligatory exercise may be associated with the persistence of eating disorder symptoms but also to treatment resistance. Therefore, assessing exercise-related attitudes and motivations may provide clinically relevant information beyond the mere frequency of physical activity.

The greater results observed with exercise commitment can be connected to the influence of eating disorders on the quality of life of suffering adolescents, resulting in negative consequences. The tendency to exercise out of obligation in patients suffering from ED has been mentioned in previous studies, e.g., Cook et al. (2015) [22]. However, this finding should be interpreted cautiously because the exercise commitment subscale demonstrated relatively low internal consistency (Cronbach’s α = 0.58) in the present sample.

The higher scores for exercise frequency and exercise for health- and social-related reasons observed among adolescents engaged in qualified sports were expected given the inclusion criteria and the training demands associated with competitive sport. Therefore, these findings should not be interpreted as independent effects of the absence of eating disorders but rather as characteristics of the comparison group. For this reason, their motivation to train could be significantly different from that of patients with eating disorders. Liu and Cao (2022) [23], while exploring how exercise motivation impacts eating disorders, reported that motivations rooted in health and social factors lead to better psychological outcomes than motivations related to weight control, which can contribute to eating disorders.

This pattern suggests that problematic exercise may be better understood through its psychological function rather than through exercise volume alone. Although adolescents engaged in qualified sports reported substantially higher exercise frequency and training volume, adolescents with eating disorders demonstrated higher levels of exercise fixation, withdrawal symptoms, and exercise for weight control. Consequently, clinicians working with adolescents with eating disorders should assess not only the amount and frequency of physical activity but also its psychological characteristics, including exercise-related motivations, compulsive exercise attitudes, feelings of guilt associated with missed exercise sessions, and withdrawal-like symptoms. Routine assessment of these psychological aspects of exercise may help identify adolescents at risk of persistent eating disorder psychopathology and support individualized treatment planning. Despite the insights gained from this study, there are several directions for future research to build upon these findings. First, the current limitations, including the partial reliability of the measurement instruments and the use of a forward-only translation procedure for the EDQ and OEQ, highlight the need for more robust methodological approaches.

In addition, several scales demonstrated internal consistency coefficients below the conventional threshold of 0.70, particularly the exercise commitment subscale. Consequently, findings based on these measures should be interpreted with caution and require replication in larger samples using psychometrically validated Polish versions of the instruments.

A further limitation is that no additional psychological constructs, such as perfectionism, compulsivity, personality traits, body image concerns, or emotion regulation difficulties, were assessed. Consequently, the present study cannot determine which psychological mechanisms may explain the observed relationship between exercise-related attitudes and behaviours and eating disorder psychopathology.

An additional limitation is the marked imbalance in gender composition between the study groups. Because gender is known to influence eating attitudes and exercise-related motivations, and because the relatively small and uneven sample precluded meaningful adjustment for gender, the observed between-group differences may partly reflect gender-related effects in addition to differences associated with eating disorder and athletic status.

A further limitation is that eating disorder status and athletic status were confounded by design. The study compared adolescents with eating disorders who did not participate in qualified sports with adolescents engaged in qualified sports who had no eating disorder diagnosis. Consequently, the independent contribution of eating disorder status to the observed differences cannot be determined. Future studies should include athletes with eating disorder symptoms and individuals with eating disorders who participate in competitive sports to disentangle the respective contributions of athletic participation and eating disorder status.

Another limitation concerns the assessment of eating disorder status in the athlete group. Athletes were excluded based on a single self-reported question regarding a previous physician diagnosis of an eating disorder, which may not have identified undiagnosed or subclinical eating disorder symptoms. Furthermore, the eligibility criteria differed between groups, as adolescents with eating disorders had a current clinical diagnosis whereas athletes were screened using a lifetime self-report item. Future studies should apply standardized screening or diagnostic procedures consistently across all study groups.

Moreover, participants received small tokens of appreciation that differed between study groups because of the different recruitment settings. Although these incentives were provided only after questionnaire completion and were not contingent on participants’ responses, their potential influence cannot be completely excluded. Future studies should consider using identical incentives across comparison groups.

Finally, the cross-sectional design precludes causal inference. Consequently, the observed associations should not be interpreted as evidence that obligatory exercise contributes to the development or maintenance of eating disorders, but rather as relationships requiring confirmation in longitudinal studies.

To ensure greater reliability and accuracy, expanding the sample size and incorporating a more diverse cohort of respondents would strengthen the generalizability of the results. Furthermore, longitudinal research could clarify the progression and causality between obligatory exercise and eating disorders while examining psychosocial traits such as perfectionism in greater depth. Comparative analyses of different sport types and their association with problematic behaviours, coupled with qualitative methods for personal insight, would enrich the findings. Finally, incorporating clinical insights into research can guide targeted treatment strategies and preventive measures and foster collaboration among clinicians, sports psychologists, and educators for better support systems.

## 5. Conclusions

This pilot study demonstrated significant differences in eating attitudes and in several psychological characteristics and motivations related to exercise between adolescents diagnosed with eating disorders and adolescents engaged in qualified sports. These findings should be interpreted in the context of the study design, as the comparison involved two populations differing not only in eating disorder status but also in athletic status and gender composition.

Key conclusions from this pilot study include the following:

- Adolescents with eating disorders demonstrated higher levels of problematic eating behaviours, exercise fixation, withdrawal symptoms, exercise commitment, and exercise for weight control than adolescents engaged in qualified sports, whereas athletes reported stronger social- and health-related motives for exercise.
- The findings suggest that the psychological characteristics of exercise, rather than exercise volume alone, may be particularly relevant when assessing adolescents with eating disorders.
- Assessment of exercise-related attitudes and motivations may provide clinically useful information that complements the evaluation of eating disorder symptoms.

The present findings suggest that health care professionals working with adolescents may benefit from assessing not only the amount of physical activity but also its psychological characteristics, including exercise-related motivations and obligatory exercise attitudes. Similarly, coaches and other professionals working with adolescents may benefit from being aware of exercise-related behaviours that could warrant further clinical assessment, particularly when accompanied by signs of disordered eating.

These findings may support more comprehensive assessment and individualized treatment planning; however, they should be interpreted in light of the study’s methodological limitations. Further research involving larger and more diverse samples is needed to confirm these findings and to clarify the independent contributions of eating disorder status, athletic participation, and gender.

## Figures and Tables

**Table 1:** Sample characteristics.

Variable	Athletes (*n* = 45)	Adolescents with ED (*n* = 45)	*p*-Value
Gender, n (%)			<0.001
Women	18/45 (40%)	33/45 (73%)	
Men	26/45 (58%)	2/45 (4.4%)	
Prefer not to answer	0	1/45 (2.2%)	
Non-binary	1/45 (2.2%)	9/45 (20%)	
Education, n (%)			0.024
University	14/45 (31%)	5/45 (11%)	
Secondary school	19/45 (42%)	20/45 (44%)	
Trade school	0	4/45 (8.9%)	
Primary school	10/45 (22%)	11/45 (24%)	
Technical secondary school	1/45 (2.2%)	5/45 (11%)	
Completed education	1/45 (2.2%)	0	
Employment, n (%)			>0.91
Occasional	12/45 (27%)	10/45 (22%)	
Permanent	3/45 (6.7%)	3/45 (6.7%)	
Not employed	30/45 (67%)	32/45 (71%)	
Weekly physical activity, n (%)			<0.001
1–2 h	0	7/45 (16%)	
2–4 h	1/45 (2.2%)	17/45 (38%)	
4–6 h	4/45 (8.9%)	16/45 (36%)	
6–8 h	10/45 (22%)	3/45 (6.7%)	
>8 h	30/45 (67%)	2/45 (4.4%)	
Organized sports classes, n (%)			0.003
No	14/45 (31%)	29/45 (64%)	
Yes	31/45 (69%)	16/45 (36%)	
Competition participation, n (%)			<0.001
No	0	45/45 (100%)	
Yes	45/45 (100%)	0	
Age (years)	16.91 ± 2.29 (17.0)	16.33 ± 1.75 (16.0)	0.43
Height (cm)	170.80 ± 9.55 (173.0)	163.67 ± 7.32 (163.0)	<0.001
Current weight (kg)	60.76 ± 12.83 (60.0)	50.56 ± 13.06 (48.0)	<0.001
Highest weight (kg)	63.32 ± 14.48 (63.0)	61.00 ± 15.01 (56.0)	0.33
Lowest weight (kg)	53.84 ± 12.67 (56.0)	44.07 ± 11.32 (42.0)	<0.001
Ideal weight (kg)	60.80 ± 12.13 (60.0)	55.50 ± 7.91 (55.0)	0.043
BMI	20.66 ± 2.97 (19.82)	18.81 ± 4.67 (17.36)	<0.001

Note: Categorical variables are presented as n (%). Continuous variables are presented as mean ± *SD* (median). Statistical significance tests used: 1. Fisher’s exact test; 2. Pearson’s chi-square test; 3. Mann–Whitney U test.

**Table 2:** Reliability of measurements used in study.

Scale	Items (*n*)	Mean (*M*)	*SD*	Cronbach’s α	McDonald’s ω
EAT	26	3.33	1.40	0.97	0.97
Exercise fixation	5	2.64	0.81	0.83	0.84
Exercise commitment	3	3.76	0.68	0.58	0.66
Withdrawal symptoms	4	4.72	1.90	0.94	0.94
Exercising for weight control	4	4.46	2.12	0.92	0.92
Interference with social life	5	4.14	1.40	0.75	0.76
Positive reward	4	4.84	1.43	0.83	0.83
Insight into problem	4	3.52	2.04	0.91	0.92
Exercise for social reasons	3	3.53	1.50	0.47	0.55
Exercise for health reasons	3	3.77	1.56	0.73	0.74
Stereotyped behaviour	2	5.08	1.80	0.82	0.82
Exercise frequency	3	3.35	0.57	0.65	0.67
Obligatory Exercise Questionnaire	10	3.27	0.48	0.69	0.74

Note: A reliability coefficient above 0.70 indicates an acceptable level of measurement accuracy for the analyzed scale. The closer the value is to 1, the higher the measurement accuracy. *n* = number of test items included in the scale; *M* = mean level of measurement scores; *SD* = standard deviation of measurement scores.

**Table 3:** The results of the Spearman correlation analysis among problematic eating behaviours (EAT-26), exercise fixation, exercise commitment, withdrawal symptoms, exercise for weight control, interference with social life, positive reward, insight into problems, exercise for social reasons, exercise for health reasons, stereotyped behaviour, exercise frequency, and the Obligatory Exercise Questionnaire (OEQ).

Variable	1	2	3	4	5	6	7	8	9	10	11	12	13
1. EAT-26	—												
2. Exercise fixation	0.77 ***	—											
3. Exercise commitment	0.51 ***	0.61 ***	—										
4. Withdrawal symptoms	0.63 ***	0.70 ***	0.57 **	—									
5. Exercise for weight control	0.82 ***	0.75 ***	0.44 **	0.61 ***	—								
6. Interference with social life	0.37 *	0.54 ***	0.41 **	0.49 ***	0.36 *	—							
7. Positive reward	0.73 ***	0.62 ***	0.44 **	0.52 ***	0.35 *	0.65 ***	—						
8. Insight into problems	0.25	0.45 **	0.64 ***	0.64 ***	0.56 **	0.64 ***	0.65 ***	—					
9. Exercise for social reasons	−0.66 ***	−0.47 ***	−0.35 *	−0.44 **	−0.58 ***	−0.30	−0.21	−0.62 ***	—				
10. Exercise for health reasons	0.08	−0.26	−0.03	−0.09	−0.28	−0.36 *	−0.29	0.02	0.51 **	—			
11. Stereotyped behaviour	−0.01	0.09	0.22	0.32	−0.09	0.32	−0.02	0.07	0.03	0.25	—		
12. Exercise frequency	0.02	0.22	0.31	0.37 *	−0.02	0.33	0.25	−0.06	0.05	0.13	0.27	—	
13. Obligatory Exercise Questionnaire	0.50 ***	0.67 ***	0.80 ***	0.71 ***	0.48 ***	0.67 ***	0.61 ***	0.23	−0.01	−0.26	0.41 **	0.63 ***	—

Note: The number of observations in the analysis was *N* = 90; * *p* < 0.05, ** *p* < 0.01, *** *p* < 0.001.

**Table 4:** The differences between the athletes and adolescents with ED are variable in terms of the levels of problematic eating behaviours (EAT-26), the Exercise Dependence Questionnaire (EDQ) subscales and the Obligatory Exercise Questionnaire (OEQ) subscales.

Dependent Variable	Athletes (*n* = 45) Mean ± *SD* (Median)	Adolescents with ED (*n* = 45) Mean ± *SD* (Median)	U	Holm-Adjusted *p*	Rank-Biserial Correlation (*rg*)
EAT-26	55.27 ± 18.37 (52.0)	117.78 ± 18.28 (124.0)	60.0	<0.001	0.94
Exercise commitment	10.78 ± 1.94 (11.0)	11.80 ± 2.02 (12.0)	679.5	0.039	0.33
Exercise fixation	10.64 ± 3.46 (10.0)	15.78 ± 2.84 (16.0)	290.5	<0.001	0.71
Exercise for health reasons	13.00 ± 4.13 (13.0)	9.64 ± 4.61 (10.0)	608.0	0.008	0.40
Exercise for social reasons	13.44 ± 3.09 (12.0)	7.71 ± 3.79 (6.0)	263.0	<0.001	0.74
Exercise frequency	10.44 ± 1.60 (11.0)	9.67 ± 1.77 (10.0)	719.5	0.079	0.29
Exercise for weight control	11.49 ± 5.28 (12.0)	24.18 ± 5.92 (27.0)	167.5	<0.001	0.83
Insight into problems	8.44 ± 4.93 (6.0)	19.73 ± 6.70 (23.0)	229.5	<0.001	0.77
Interference with social life	19.24 ± 7.00 (21.0)	22.11 ± 6.76 (24.0)	750.5	0.138	0.26
Obligatory Exercise Questionnaire	32.02 ± 4.90 (32.0)	33.33 ± 4.62 (34.0)	756.0	0.138	0.25
Positive reward	18.53 ± 5.91 (18.0)	20.20 ± 5.45 (20.0)	802.5	0.180	0.21
Stereotyped behaviour	10.73 ± 2.63 (11.0)	9.58 ± 4.31 (12.0)	944.0	0.575	0.07
Withdrawal symptoms	15.53 ± 7.60 (17.0)	22.22 ± 6.02 (24.0)	486.5	<0.001	0.52

Note: Data are presented as mean ± *SD* (median). *p*-values are Holm-adjusted. Effect size is reported as the rank-biserial correlation (*rg*).

## Data Availability

Data from this study are available: https://osf.io/u4pa2/?view_only=919085578e894696b38da5f5bfc743dc (accessed on 23 May 2026).

## Supplementary Materials

The following supporting information can be downloaded at https://www.mdpi.com/article/10.3390/jcm15145455/s1, Table S1: Comparison of Spearman correlation coefficients between athletes and adolescents with eating disorders; Figure S1: Correlations between components expressing the measurement EAT; Figure S2: Correlations between components expressing the measurement Exercise fixation; Figure S3: Correlations between components expressing the measurement Exercise commitment; Figure S4: Correlations between components expressing the measurement Withdrawal symptoms; Figure S5: Correlations between components expressing the measurement Exercising for weight control; Figure S6: Correlations between components expressing the measurement Interference with social life; Figure S7: Correlations between components expressing the measurement Positive reward; Figure S8: Correlations between components expressing the measurement Insight into problem; Figure S9: Correlations between components expressing the measurement Exercise for social reasons, Figure S10: Correlations between components expressing the measurement Exercise for health reasons; Figure S11: Correlations between components expressing the measurement Stereotyped behaviour; Figure S12: Correlations between components expressing the measurement Exercise frequency; Figure S13: Correlations between components expressing the measurement Obligatory Exercise Questionnaire; Table S2: Diagnostics of normality of distributions in the entire sample; Table S3: Normality diagnostics of distributions within subgroups; Table S4: Equality of variance diagnostics; Figure S14: Differences between groups in terms of the level of the variable EAT; Figure S15: Differences between groups in terms of the level of the variable Exercise commitment; Figure S16: Differences between groups in terms of the level of the variable Exercise fixation; Figure S17: Differences between groups in terms of the level of the variable Exercise for health reasons; Figure S18: Differences between groups in terms of the level of the variable Exercise for social reasons, Figure S19: Differences between groups in terms of the level of the variable Exercise frequency; Figure S20: Differences between groups in terms of the level of the variable Exercising for weight control; Figure S21: Differences between groups in terms of the level of the variable Insight into problem; Figure S22: Differences between groups in terms of the level of the variable Interference with social life; Figure S23: Differences between groups in terms of the level of the variable Obligatory Exercise Questionnaire; Figure S24: Differences between groups in terms of the level of the variable Positive reward; Figure S25: Differences between groups in terms of the level of the variable Stereotyped behaviour; Figure S26: Differences between groups in terms of the level of the variable withdrawal symptoms.

## Abbreviations

The following abbreviations are used in this manuscript:

OEQ	Obligatory Exercise Questionnaire
EDQ	Exercise Dependence Questionnaire
EAT-26	Eating Attitudes Test
ED	Eating Disorders
EA	Exercise Addiction
IPAO	Inventory of Physical Activity Objectives
