# Supplementary material for: Obligatory Exercise and Eating Attitudes—A Pilot Study on Polish Adolescents
Source: J Clin Med. 2026 Jul 12;15(14):5455. doi: 10.3390/jcm15145455 (PMC13412378; doi:10.3390/jcm15145455)
Supplement: Supplementary file 1 [file jcm-15-05455-s001.zip › jcm-4367033-supplementary.pdf]

Supplementary Materials

Table S1. Comparison of Spearman correlation coefficients between athletes and adolescents with eating disorders.

Variable 1	Variable 2	Athletes r (95% CI)	Adolescents with ED r (95% CI)	Fisher's z
**EAT**	**Exercise fixation**	0.63*** (0.42,0.78)	0.3 (0.01,0.55)	2
**EAT**	**Exercise commitment**	0.52* (0.26,0.70)	0.38 (0.10,0.61)	0.77
**Exercise fixation**	**Exercise commitment**	0.69*** (0.50,0.82)	0.41 (0.14,0.63)	1.87
**EAT**	**Withdrawal symptoms**	0.38 (0.10,0.61)	0.51* (0.25,0.70)	-0.73
**Exercise fixation**	**Withdrawal symptoms**	0.64*** (0.43,0.79)	0.45 (0.18,0.66)	1.28
**Exercise commitment**	**Withdrawal symptoms**	0.54** (0.29,0.72)	0.46 (0.19,0.66)	0.51
**EAT**	**Exercising for weight control**	0.58** (0.35,0.75)	0.34 (0.05,0.57)	1.46
**Exercise fixation**	**Exercising for weight control**	0.44 (0.17,0.65)	0.23 (-0.07,0.49)	1.1
**Exercise commitment**	**Exercising for weight control**	0.45 (0.18,0.66)	0.05 (-0.25,0.34)	2.01
**Withdrawal symptoms**	**Exercising for weight control**	0.32 (0.03,0.56)	0.35 (0.07,0.59)	-0.18
**EAT**	**Interference with social life**	0.49* (0.23,0.68)	0.33 (0.04,0.57)	0.87
**Exercise fixation**	**Interference with social life**	0.64*** (0.43,0.79)	0.3 (0.01,0.55)	2.04
**Exercise commitment**	**Interference with social life**	0.68*** (0.49,0.81)	0.59** (0.36,0.75)	0.75
**Withdrawal symptoms**	**Interference with social life**	0.58** (0.35,0.75)	0.56** (0.32,0.73)	0.13
**Exercising for weight control**	**Interference with social life**	0.47* (0.21,0.67)	0.01 (-0.28,0.30)	2.31
**EAT**	**Positive reward**	0.44 (0.17,0.65)	0.18 (-0.12,0.45)	1.35
**Exercise fixation**	**Positive reward**	0.56** (0.32,0.74)	0.29 (0.00,0.54)	1.55
**Exercise commitment**	**Positive reward**	0.46 (0.20,0.67)	0.2 (-0.10,0.47)	1.35
**Withdrawal symptoms**	**Positive reward**	0.69*** (0.50,0.82)	0.4 (0.12,0.62)	1.98
**Exercising for weight control**	**Positive reward**	0.48* (0.21,0.68)	0.54* (0.29,0.72)	-0.35
**Interference with social life**	**Positive reward**	0.52* (0.26,0.70)	-0.03 (-0.32,0.26)	2.76
**EAT**	**Insight into problem**	0.34 (0.05,0.57)	0.4 (0.12,0.62)	-0.32
**Exercise fixation**	**Insight into problem**	0.48* (0.22,0.68)	0.09 (-0.20,0.38)	1.98
**Exercise commitment**	**Insight into problem**	0.41 (0.13,0.63)	0.27 (-0.02,0.52)	0.71
**Withdrawal symptoms**	**Insight into problem**	0.35 (0.06,0.58)	0.46 (0.19,0.66)	-0.62
**Exercising for weight control**	**Insight into problem**	0.24 (-0.06,0.50)	0.17 (-0.13,0.44)	0.32
**Interference with social life**	**Insight into problem**	0.48* (0.21,0.68)	0.55** (0.31,0.73)	-0.46
**Positive reward**	**Insight into problem**	0.1 (-0.20,0.39)	0.05 (-0.24,0.34)	0.23
**EAT**	**Exercise for social reasons**	-0.07 (-0.36,0.22)	-0.46 (-0.67,-0.20)	1.96
**Exercise fixation**	**Exercise for social reasons**	-0.06 (-0.35,0.24)	-0.14 (-0.42,0.16)	0.36
**Exercise commitment**	**Exercise for social reasons**	-0.1 (-0.38,0.20)	-0.25 (-0.51,0.04)	0.72
**Withdrawal symptoms**	**Exercise for social reasons**	-0.06 (-0.35,0.24)	-0.41 (-0.63,-0.13)	1.74
**Exercising for weight control**	**Exercise for social reasons**	0.31 (0.01,0.55)	-0.57** (-0.74,-0.33)	4.39***
**Interference with social life**	**Exercise for social reasons**	0.17 (-0.13,0.44)	-0.44 (-0.65,-0.17)	2.94
**Positive reward**	**Exercise for social reasons**	0.04 (-0.26,0.33)	-0.27 (-0.52,0.03)	1.41
**Insight into problem**	**Exercise for social reasons**	-0.01 (-0.30,0.28)	-0.58** (-0.74,-0.34)	2.97
**EAT**	**Exercise for health reasons**	-0.02 (-0.31,0.27)	-0.18 (-0.45,0.12)	0.75
**Exercise fixation**	**Exercise for health reasons**	-0.05 (-0.34,0.25)	-0.14 (-0.41,0.16)	0.39
**Exercise commitment**	**Exercise for health reasons**	-0.17 (-0.44,0.13)	0.21 (-0.08,0.48)	-1.8
**Withdrawal symptoms**	**Exercise for health reasons**	0.17 (-0.13,0.44)	0.07 (-0.23,0.35)	0.48
**Exercising for weight control**	**Exercise for health reasons**	0.4 (0.12,0.62)	-0.31 (-0.55,-0.02)	3.39
**Interference with social life**	**Exercise for health reasons**	0.12 (-0.18,0.40)	0.28 (-0.02,0.53)	-0.77
**Positive reward**	**Exercise for health reasons**	0.22 (-0.08,0.48)	-0.04 (-0.33,0.25)	1.23
**Insight into problem**	**Exercise for health reasons**	-0.2 (-0.47,0.10)	0.02 (-0.27,0.31)	-1.05
**Exercise for social reasons**	**Exercise for health reasons**	0.42 (0.15,0.64)	0.32 (0.03,0.56)	0.57
**EAT**	**Stereotyped behaviour**	0.05 (-0.25,0.34)	0.02 (-0.28,0.31)	0.13
**Exercise fixation**	**Stereotyped behaviour**	0.05 (-0.24,0.34)	0.2 (-0.10,0.47)	-0.68
**Exercise commitment**	**Stereotyped behaviour**	0.06 (-0.24,0.35)	0.42 (0.14,0.63)	-1.76
**Withdrawal symptoms**	**Stereotyped behaviour**	0.34 (0.06,0.58)	0.42 (0.14,0.63)	-0.38

**Exercising for weight control**	**Stereotyped behaviour**	-0.21 (-0.48,0.09)	-0.15 (-0.43,0.15)	-0.3
**Interference with social life**	**Stereotyped behaviour**	0.15 (-0.15,0.42)	0.49* (0.23,0.69)	-1.78
**Positive reward**	**Stereotyped behaviour**	-0.08 (-0.36,0.22)	0.07 (-0.23,0.35)	-0.66
**Insight into problem**	**Stereotyped behaviour**	0.12 (-0.18,0.40)	0.09 (-0.21,0.37)	0.13
**Exercise for social reasons**	**Stereotyped behaviour**	-0.02 (-0.31,0.27)	-0.04 (-0.33,0.26)	0.06
**Exercise for health reasons**	**Stereotyped behaviour**	0.04 (-0.26,0.33)	0.49* (0.23,0.69)	-2.29
**EAT**	**Exercise frequency**	0.52* (0.26,0.70)	0.34 (0.05,0.58)	1.01
**Exercise fixation**	**Exercise frequency**	0.54** (0.29,0.72)	0.49* (0.23,0.69)	0.28
**Exercise commitment**	**Exercise frequency**	0.53* (0.27,0.71)	0.36 (0.07,0.59)	0.95
**Withdrawal symptoms**	**Exercise frequency**	0.62*** (0.40,0.77)	0.49* (0.23,0.68)	0.9
**Exercising for weight control**	**Exercise frequency**	0.44 (0.17,0.65)	0.04 (-0.25,0.33)	1.98
**Interference with social life**	**Exercise frequency**	0.36 (0.08,0.59)	0.49* (0.23,0.69)	-0.72
**Positive reward**	**Exercise frequency**	0.52* (0.26,0.70)	0.05 (-0.25,0.34)	2.38
**Insight into problem**	**Exercise frequency**	0 (-0.29,0.30)	0.32 (0.03,0.56)	-1.48
**Exercise for social reasons**	**Exercise frequency**	-0.18 (-0.45,0.12)	-0.24 (-0.50,0.05)	0.32
**Exercise for health reasons**	**Exercise frequency**	0.05 (-0.25,0.33)	0.1 (-0.20,0.38)	-0.24
**Stereotyped behaviour**	**Exercise frequency**	0.08 (-0.22,0.37)	0.45 (0.18,0.66)	-1.86
**EAT**	**Obligatory Exercise Questionnaire**	0.63*** (0.41,0.78)	0.37 (0.09,0.60)	1.62
**Exercise fixation**	**Obligatory Exercise Questionnaire**	0.74*** (0.57,0.85)	0.52* (0.27,0.71)	1.71
**Exercise commitment**	**Obligatory Exercise Questionnaire**	0.79*** (0.64,0.88)	0.80*** (0.67,0.89)	-0.22
**Withdrawal symptoms**	**Obligatory Exercise Questionnaire**	0.68*** (0.48,0.81)	0.50* (0.24,0.69)	1.28
**Exercising for weight control**	**Obligatory Exercise Questionnaire**	0.64*** (0.42,0.78)	0.04 (-0.26,0.33)	3.29
**Interference with social life**	**Obligatory Exercise Questionnaire**	0.70*** (0.52,0.83)	0.61*** (0.39,0.77)	0.72
**Positive reward**	**Obligatory Exercise Questionnaire**	0.76*** (0.60,0.86)	0.16 (-0.14,0.44)	3.84**
**Insight into problem**	**Obligatory Exercise Questionnaire**	0.32 (0.03,0.56)	0.34 (0.05,0.57)	-0.08
**Exercise for social reasons**	**Obligatory Exercise Questionnaire**	-0.07 (-0.35,0.23)	-0.27 (-0.53,0.02)	0.98
**Exercise for health reasons**	**Obligatory Exercise Questionnaire**	0.01 (-0.29,0.30)	0.18 (-0.12,0.45)	-0.81
**Stereotyped behaviour**	**Obligatory Exercise Questionnaire**	-0.04 (-0.33,0.25)	0.52* (0.26,0.70)	-2.82
**Exercise frequency**	**Obligatory Exercise Questionnaire**	0.75*** (0.58,0.85)	0.73*** (0.56,0.84)	0.16

Note: r = Spearman's rank correlation coefficient; CI = 95% confidence interval; z = Fisher's z statistic for comparing correlation coefficients between groups. Asterisks denote statistical significance of the corresponding statistic: * $p < 0.05$, ** $p < 0.01$, *** $p < 0.001$.

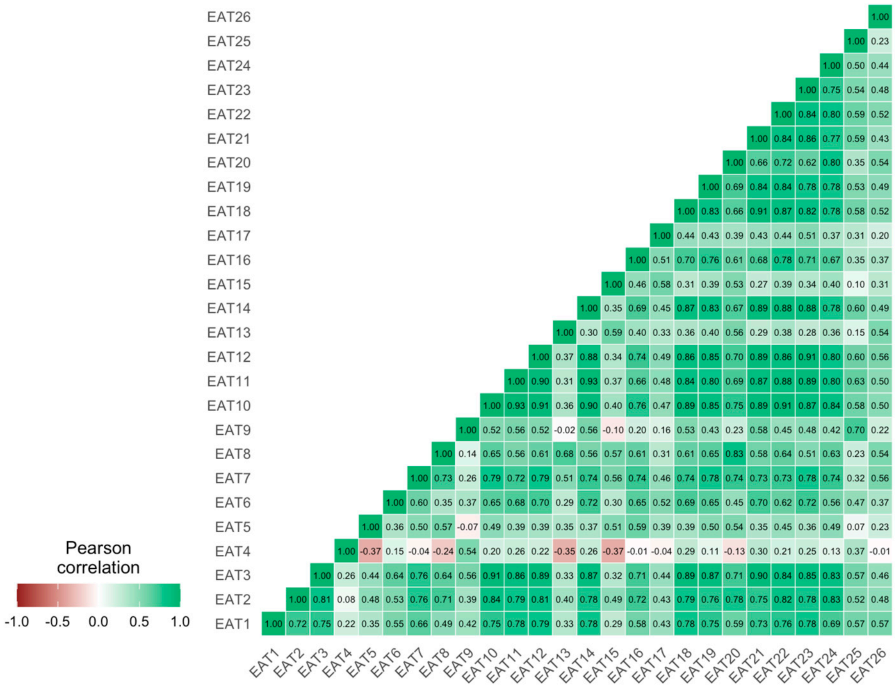

Figure S1. Correlations between components expressing the measurement EAT.

Note: Figure presents estimates of Pearson correlation coefficients. The closer to 1 (the greater the green saturation), the stronger the association between test components. Diagonal values of 1 indicate a component's correlation with itself.

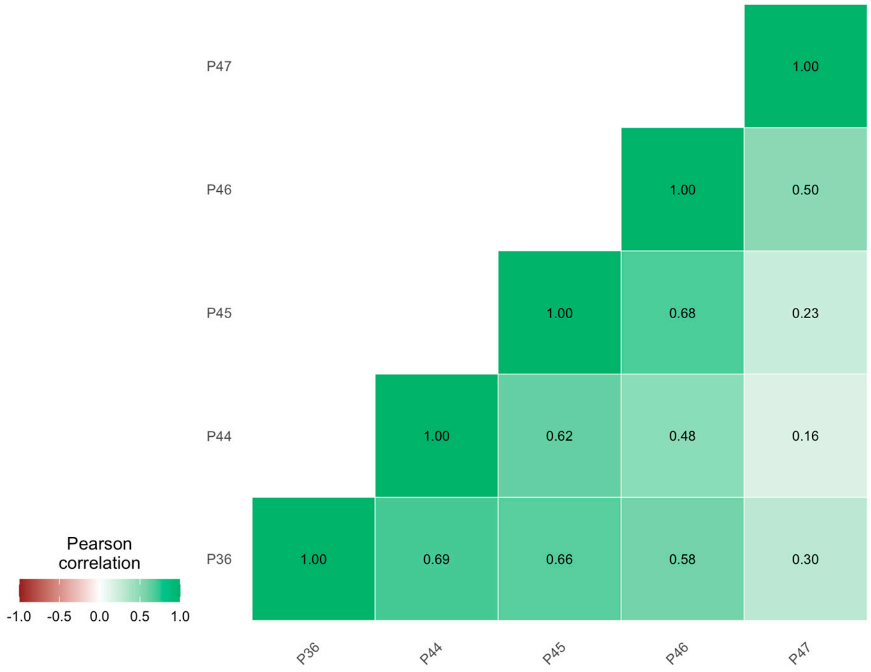

Figure S2. Correlations between components expressing the measurement Exercise fixation.

Note: Figure presents estimates of Pearson correlation coefficients. The closer to 1 (the greater the green saturation), the stronger the association between test components. Diagonal values of 1 indicate a component's correlation with itself.

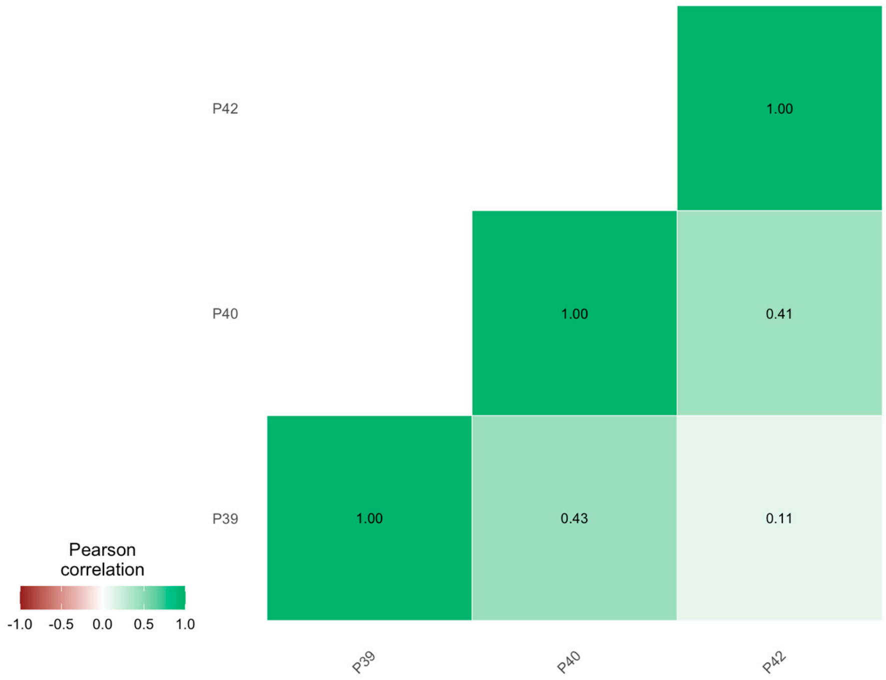

Figure S3. Correlations between components expressing the measurement Exercise commitment.

Note: Figure presents estimates of Pearson correlation coefficients. The closer to 1 (the greater the green saturation), the stronger the association between test components. Diagonal values of 1 indicate a component's correlation with itself.

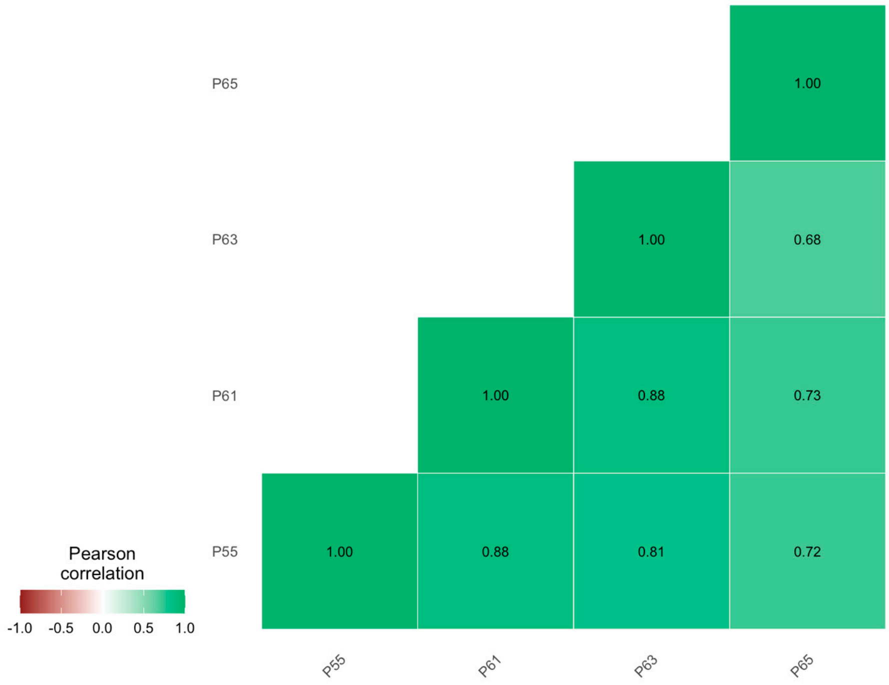

Figure S4. Correlations between components expressing the measurement Withdrawal symptoms.

Note: Figure presents estimates of Pearson correlation coefficients. The closer to 1 (the greater the green saturation), the stronger the association between test components. Diagonal values of 1 indicate a component's correlation with itself.

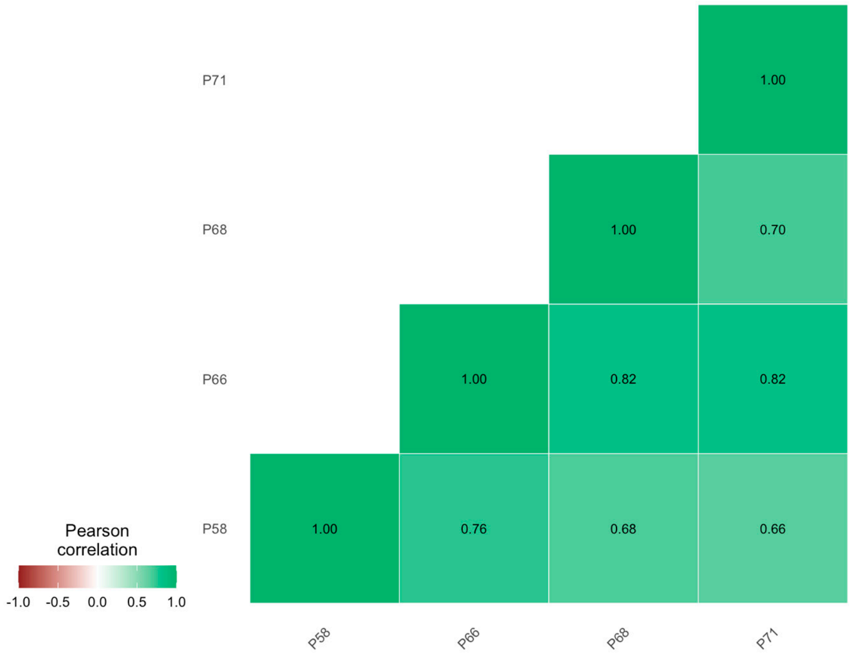

Figure S5. Correlations between components expressing the measurement Exercising for weight control.

Note: Figure presents estimates of Pearson correlation coefficients. The closer to 1 (the greater the green saturation), the stronger the association between test components. Diagonal values of 1 indicate a component's correlation with itself.

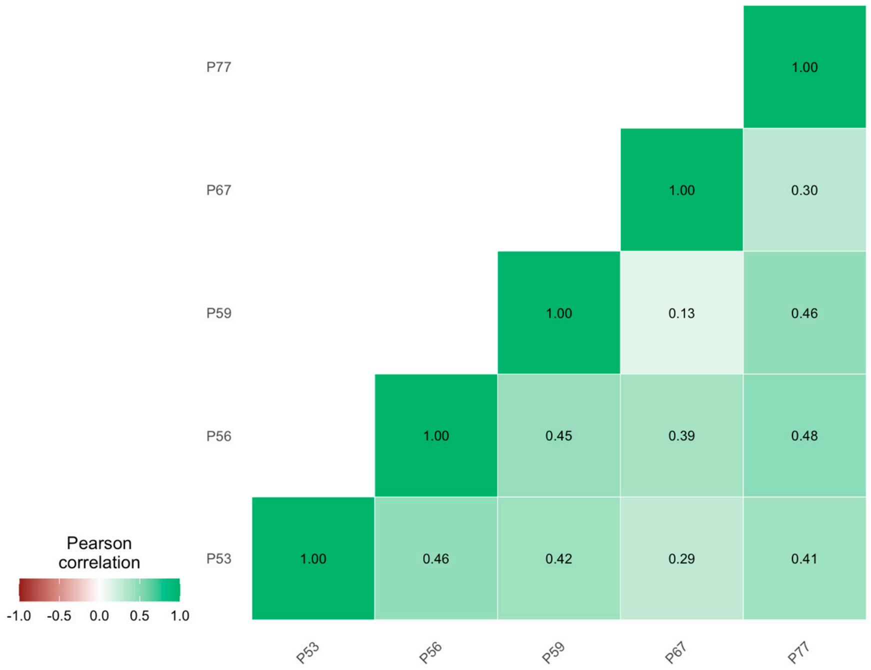

Figure S6. Correlations between components expressing the measurement Interference with social life.

Note: Figure presents estimates of Pearson correlation coefficients. The closer to 1 (the greater the green saturation), the stronger the association between test components. Diagonal values of 1 indicate a component's correlation with itself.

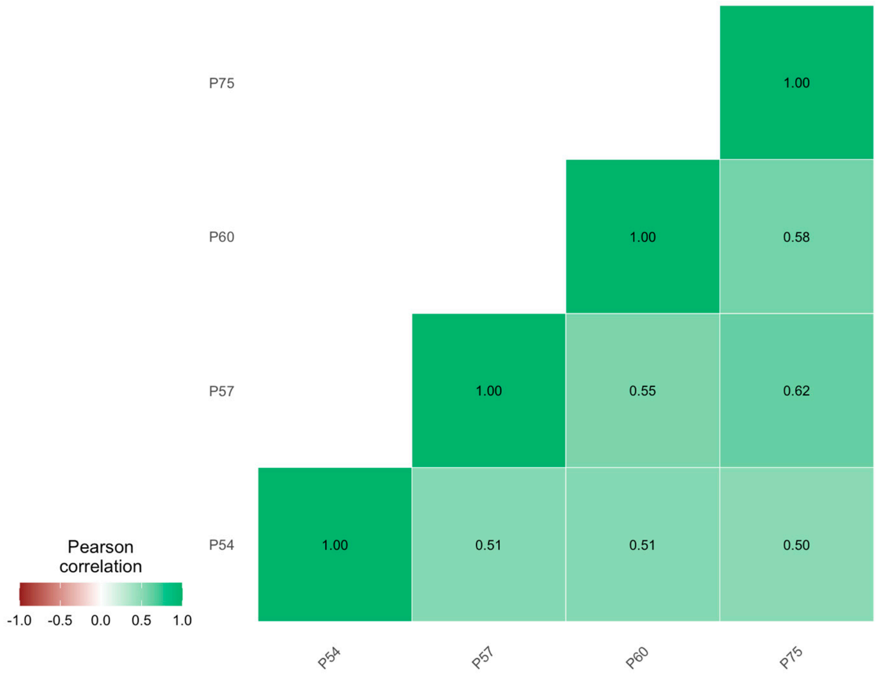

Figure S7. Correlations between components expressing the measurement Positive reward.

Note: Figure presents estimates of Pearson correlation coefficients. The closer to 1 (the greater the green saturation), the stronger the association between test components. Diagonal values of 1 indicate a component's correlation with itself.

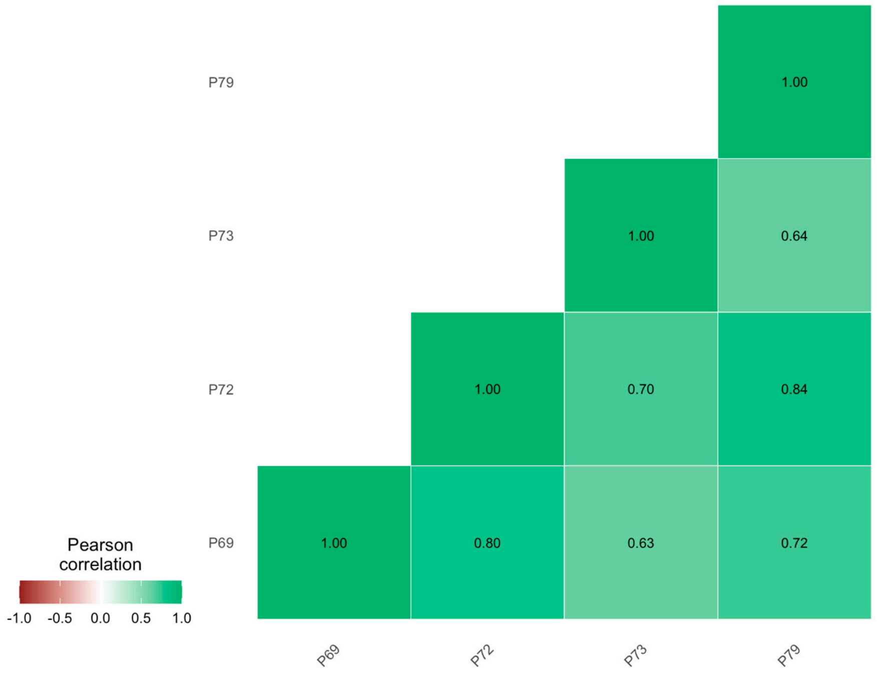

Figure S8. Correlations between components expressing the measurement Insight into problem.

Note: Figure presents estimates of Pearson correlation coefficients. The closer to 1 (the greater the green saturation), the stronger the association between test components. Diagonal values of 1 indicate a component's correlation with itself.

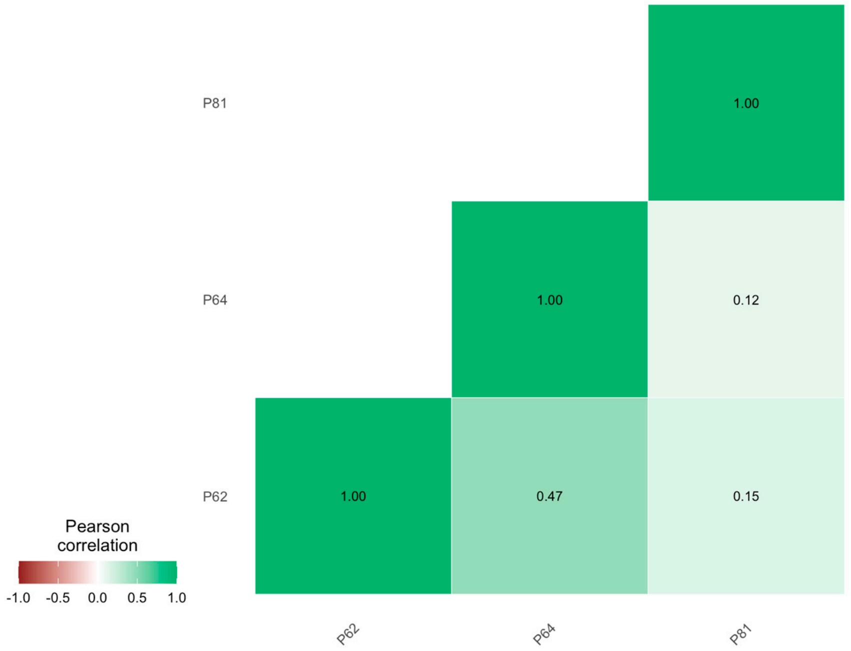

Figure S9. Correlations between components expressing the measurement Exercise for social reasons.

Note: Figure presents estimates of Pearson correlation coefficients. The closer to 1 (the greater the green saturation), the stronger the association between test components. Diagonal values of 1 indicate a component's correlation with itself.

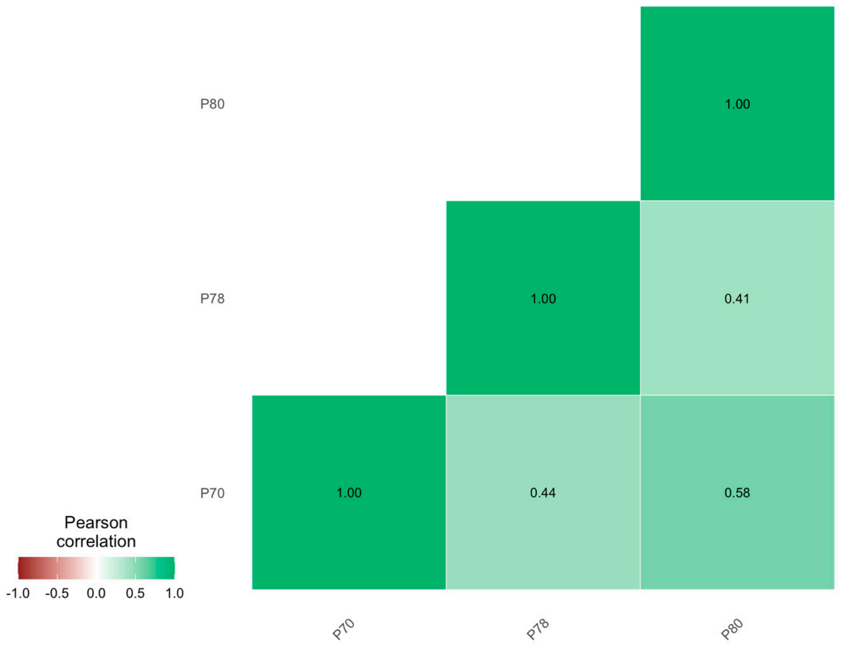

Figure S10. Correlations between components expressing the measurement Exercise for health reasons.

Note: Figure presents estimates of Pearson correlation coefficients. The closer to 1 (the greater the green saturation), the stronger the association between test components. Diagonal values of 1 indicate a component's correlation with itself.

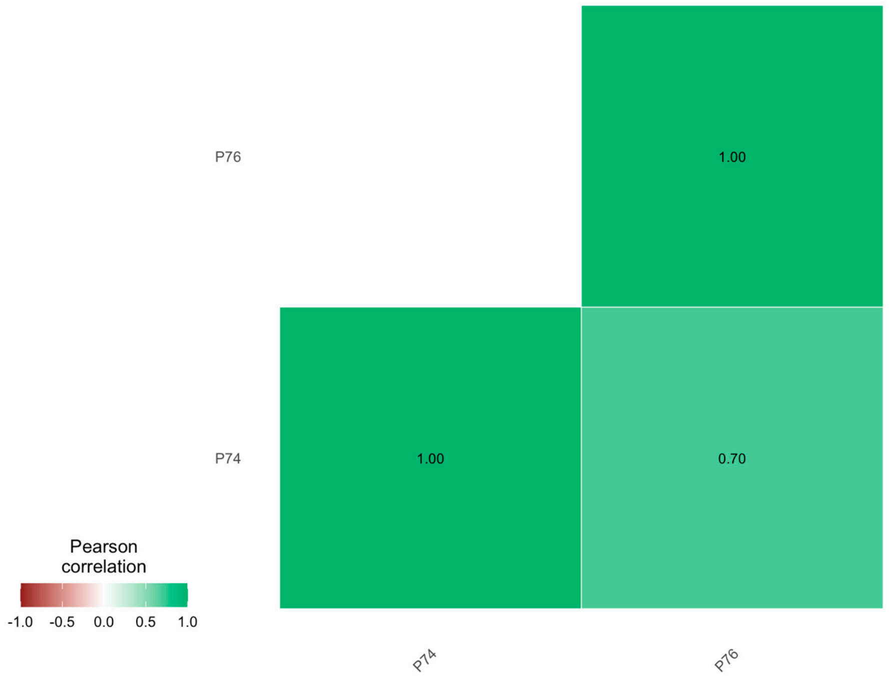

Figure S11. Correlations between components expressing the measurement Stereotyped behaviour.

Note: Figure presents estimates of Pearson correlation coefficients. The closer to 1 (the greater the green saturation), the stronger the association between test components. Diagonal values of 1 indicate a component's correlation with itself.

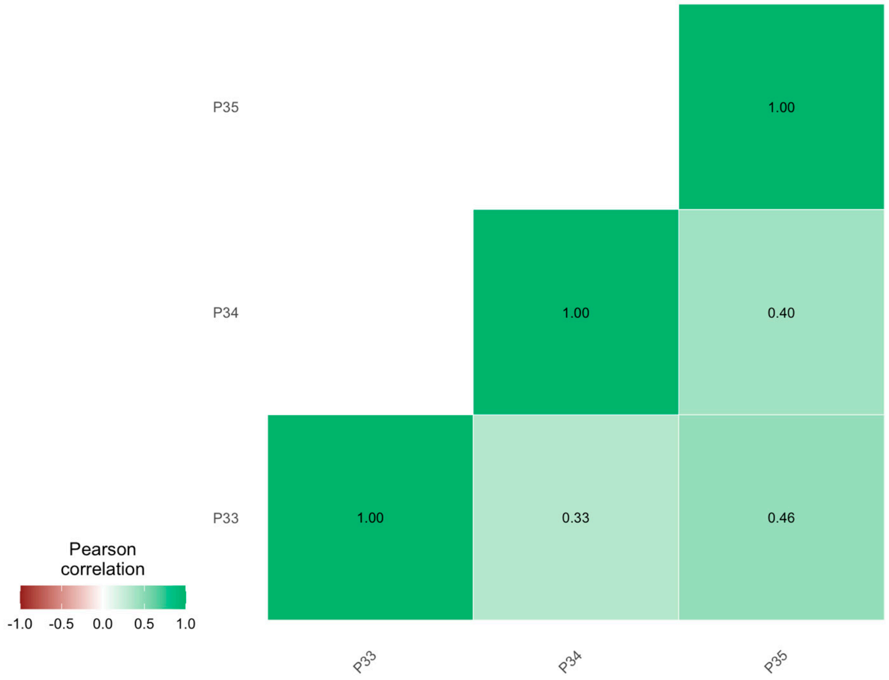

Figure S12. Correlations between components expressing the measurement Exercise frequency.

Note: Figure presents estimates of Pearson correlation coefficients. The closer to 1 (the greater the green saturation), the stronger the association between test components. Diagonal values of 1 indicate a component's correlation with itself.

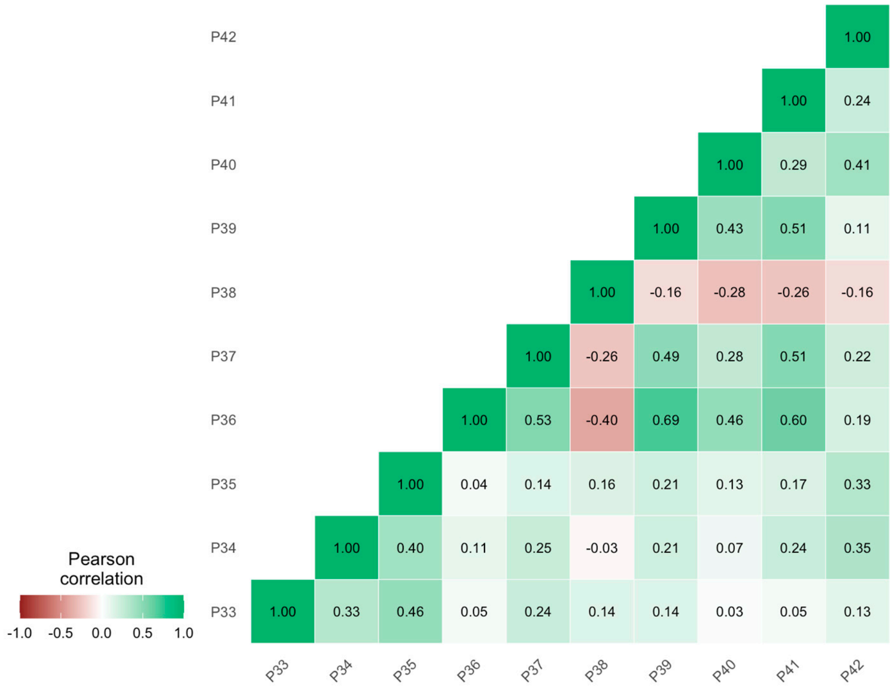

Figure S13. Correlations between components expressing the measurement Obligatory Exercise Questionnaire.

Note: Figure presents estimates of Pearson correlation coefficients. The closer to 1 (the greater the green saturation), the stronger the association between test components. Diagonal values of 1 indicate a component's correlation with itself.

Table S2. Diagnostics of normality of distributions in the entire sample.

Variable	Test	d	*p*	Results
EAT	Kołmogorow-Smirnow-Lilliefors	0.15	< 0.001	the distribution deviates from the normal
Exercise fixation	Kołmogorow-Smirnow-Lilliefors	0.159	< 0.001	the distribution deviates from the normal
Exercise commitment	Kołmogorow-Smirnow-Lilliefors	0.17	< 0.001	the distribution deviates from the normal
Withdrawal symptoms	Kołmogorow-Smirnow-Lilliefors	0.162	< 0.001	the distribution deviates from the normal
Exercising for weight control	Kołmogorow-Smirnow-Lilliefors	0.173	< 0.001	the distribution deviates from the normal
Interference with social life	Kołmogorow-Smirnow-Lilliefors	0.119	0.004	the distribution deviates from the normal
Positive reward	Kołmogorow-Smirnow-Lilliefors	0.088	0.082	no basis for rejecting normality
Insight into problem	Kołmogorow-Smirnow-Lilliefors	0.159	< 0.001	the distribution deviates from the normal
Exercise for social reasons	Kołmogorow-Smirnow-Lilliefors	0.113	0.006	the distribution deviates from the normal
Exercise for health reasons	Kołmogorow-Smirnow-Lilliefors	0.084	0.121	no basis for rejecting normality
Stereotyped behaviour	Kołmogorow-Smirnow-Lilliefors	0.237	< 0.001	the distribution deviates from the normal
Exercise frequency	Kołmogorow-Smirnow-Lilliefors	0.165	< 0.001	the distribution deviates from the normal
Obligatory Exercise Questionnaire	Kołmogorow-Smirnow-Lilliefors	0.118	0.004	the distribution deviates from the normal

Table S3. Normality diagnostics of distributions within subgroups.

Dependent variable	Group	Test	SW	*p*	Results
EAT	Athletes	Shapiro-Wilk	0.888	< 0.001	the distribution deviates from the normal
EAT	Adolescents with ED	Shapiro-Wilk	0.836	< 0.001	the distribution deviates from the normal
Exercise fixation	Athletes	Shapiro-Wilk	0.949	0.047	the distribution deviates from the normal
Exercise fixation	Adolescents with ED	Shapiro-Wilk	0.81	< 0.001	the distribution deviates from the normal
Exercise commitment	Athletes	Shapiro-Wilk	0.945	0.032	the distribution deviates from the normal
Exercise commitment	Adolescents with ED	Shapiro-Wilk	0.858	< 0.001	the distribution deviates from the normal
Withdrawal symptoms	Athletes	Shapiro-Wilk	0.944	0.03	the distribution deviates from the normal
Withdrawal symptoms	Adolescents with ED	Shapiro-Wilk	0.752	< 0.001	the distribution deviates from the normal
Exercising for weight control	Athletes	Shapiro-Wilk	0.948	0.042	the distribution deviates from the normal
Exercising for weight control	Adolescents with ED	Shapiro-Wilk	0.638	< 0.001	the distribution deviates from the normal
Interference with social life	Athletes	Shapiro-Wilk	0.967	0.216	no basis for rejecting normality
Interference with social life	Adolescents with ED	Shapiro-Wilk	0.918	0.004	the distribution deviates from the normal
Positive reward	Athletes	Shapiro-Wilk	0.938	0.018	the distribution deviates from the normal
Positive reward	Adolescents with ED	Shapiro-Wilk	0.912	0.002	the distribution deviates from the normal
Insight into problem	Athletes	Shapiro-Wilk	0.842	< 0.001	the distribution deviates from the normal
Insight into problem	Adolescents with ED	Shapiro-Wilk	0.832	< 0.001	the distribution deviates from the normal
Exercise for social reasons	Athletes	Shapiro-Wilk	0.959	0.116	no basis for rejecting normality
Exercise for social reasons	Adolescents with ED	Shapiro-Wilk	0.892	< 0.001	the distribution deviates from the normal
Exercise for health reasons	Athletes	Shapiro-Wilk	0.974	0.396	no basis for rejecting normality
Exercise for health reasons	Adolescents with ED	Shapiro-Wilk	0.949	0.047	the distribution deviates from the normal
Stereotyped behaviour	Athletes	Shapiro-Wilk	0.898	< 0.001	the distribution deviates from the normal
Stereotyped behaviour	Adolescents with ED	Shapiro-Wilk	0.742	< 0.001	the distribution deviates from the normal
Exercise frequency	Athletes	Shapiro-Wilk	0.843	< 0.001	the distribution deviates from the normal
Exercise frequency	Adolescents with ED	Shapiro-Wilk	0.849	< 0.001	the distribution deviates from the normal
Obligatory Exercise Questionnaire	Athletes	Shapiro-Wilk	0.978	0.543	no basis for rejecting normality
Obligatory Exercise Questionnaire	Adolescents with ED	Shapiro-Wilk	0.831	< 0.001	the distribution deviates from the normal

Table S4. Equality of variance diagnostics.

Dependent variable	Test	var	*p*	Results
EAT	Levene	0.015	0.904	no grounds for rejecting the equality of variances
Exercise fixation	Levene	4.466	0.037	unequal variances
Exercise commitment	Levene	0.271	0.604	no grounds for rejecting the equality of variances
Withdrawal symptoms	Levene	6.538	0.012	unequal variances
Exercising for weight control	Levene	0.205	0.652	no grounds for rejecting the equality of variances
Interference with social life	Levene	0.666	0.417	no grounds for rejecting the equality of variances
Positive reward	Levene	1.473	0.228	no grounds for rejecting the equality of variances
Insight into problem	Levene	5.239	0.024	unequal variances
Exercise for social reasons	Levene	1.249	0.267	no grounds for rejecting the equality of variances
Exercise for health reasons	Levene	1.058	0.307	no grounds for rejecting the equality of variances
Stereotyped behaviour	Levene	13.743	< 0.001	unequal variances
Exercise frequency	Levene	0.017	0.898	no grounds for rejecting the equality of variances
Obligatory Exercise Questionnaire	Levene	0.812	0.37	no grounds for rejecting the equality of variances

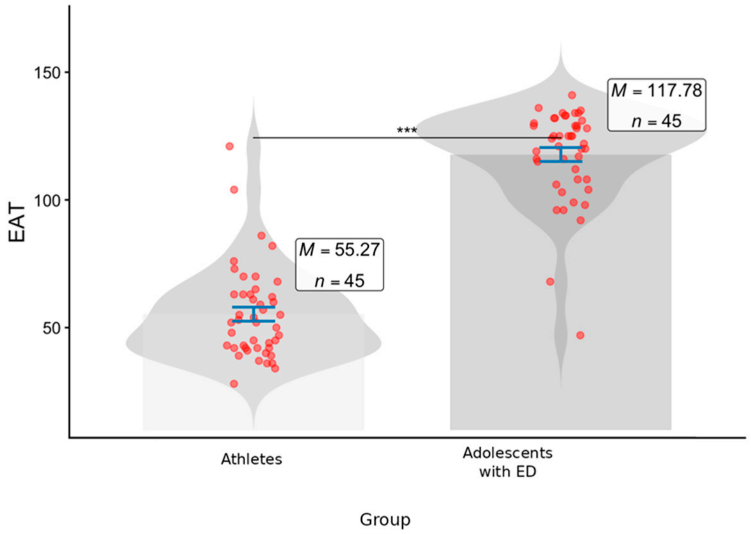

Figure S14. Differences between groups in terms of the level of the variable EAT.

Note: * . p < .05, ** . p < .01, *** . p < .001, ni - insignificant result. Blue error whiskers indicate the standard error of the mean (SE). Red dots indicate the values of individual observations.

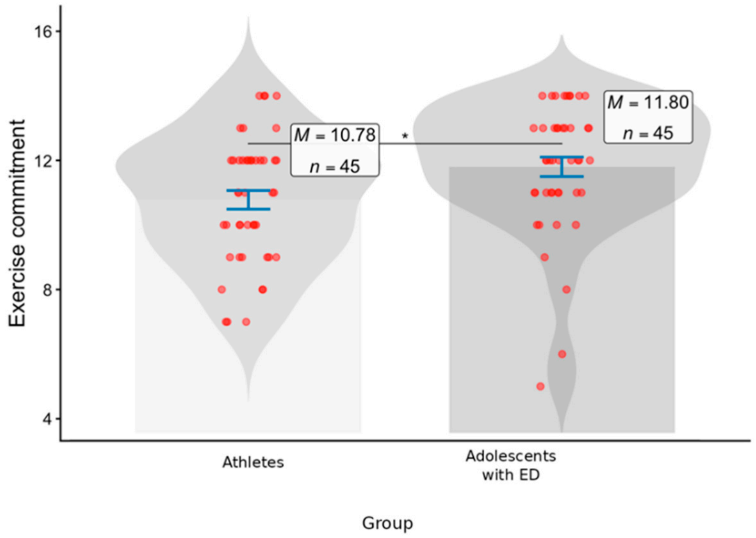

Figure S15. Differences between groups in terms of the level of the variable Exercise commitment.

Note: * . *p* < .05, ** . *p* < .01, *** . *p* < .001, *ni - insignificant result*. Blue error whiskers indicate the standard error of the mean (SE). Red dots indicate the values of individual observations.

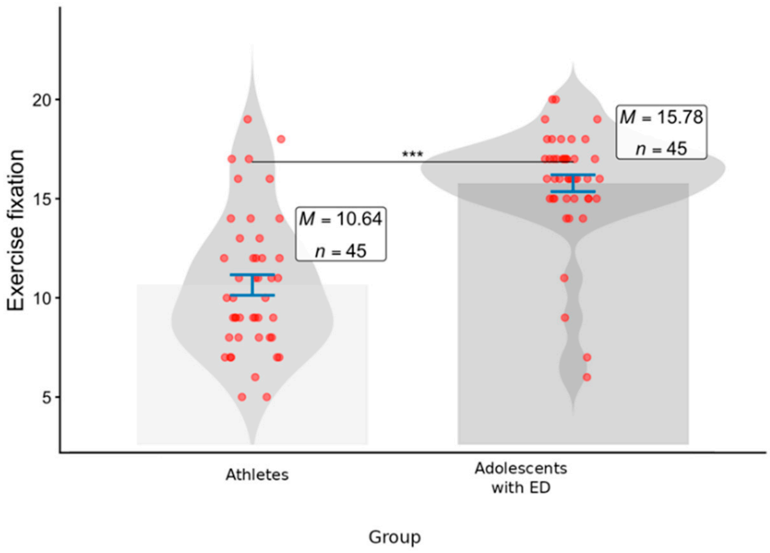

Figure S16. Differences between groups in terms of the level of the variable Exercise fixation.

Note: * . $p < .05$, ** . $p < .01$, *** . $p < .001$, *ni - insignificant result.* Blue error whiskers indicate the standard error of the mean (SE). Red dots indicate the values of individual observations.

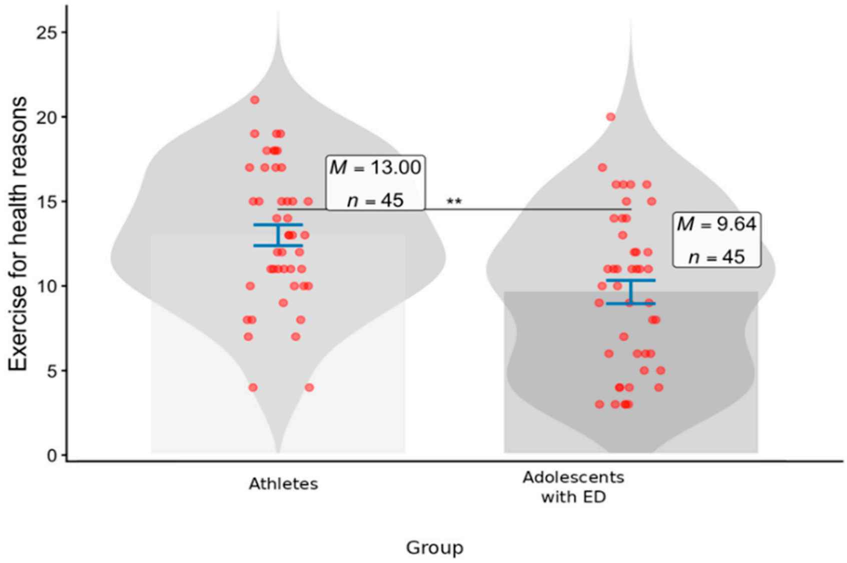

Figure S17. Differences between groups in terms of the level of the variable Exercise for health reasons.

Note: * . $p < .05$, ** . $p < .01$, *** . $p < .001$, *ni - insignificant result.* Blue error whiskers indicate the standard error of the mean (SE). Red dots indicate the values of individual observations.

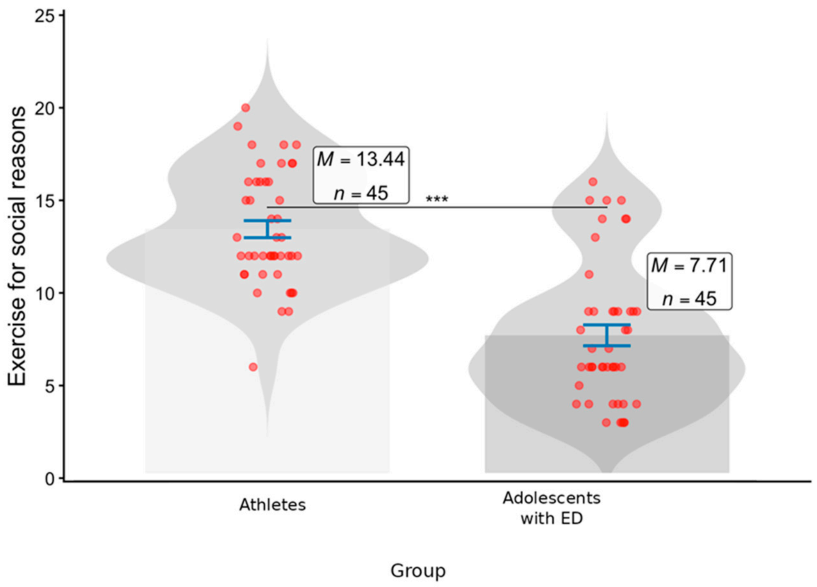

Figure S18. Differences between groups in terms of the level of the variable Exercise for social reasons.

Note: * . $p < .05$, ** . $p < .01$, *** . $p < .001$, *ni - insignificant result*. Blue error whiskers indicate the standard error of the mean (SE). Red dots indicate the values of individual observations.

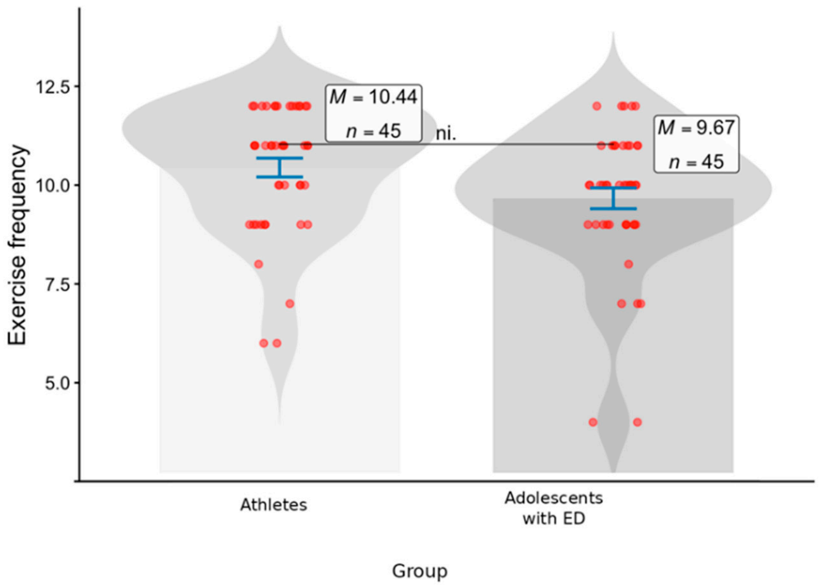

Figure S19. Differences between groups in terms of the level of the variable Exercise frequency.

Note: * . $p < .05$, ** . $p < .01$, *** . $p < .001$, *ni - insignificant result*. Blue error whiskers indicate the standard error of the mean (SE). Red dots indicate the values of individual observations.

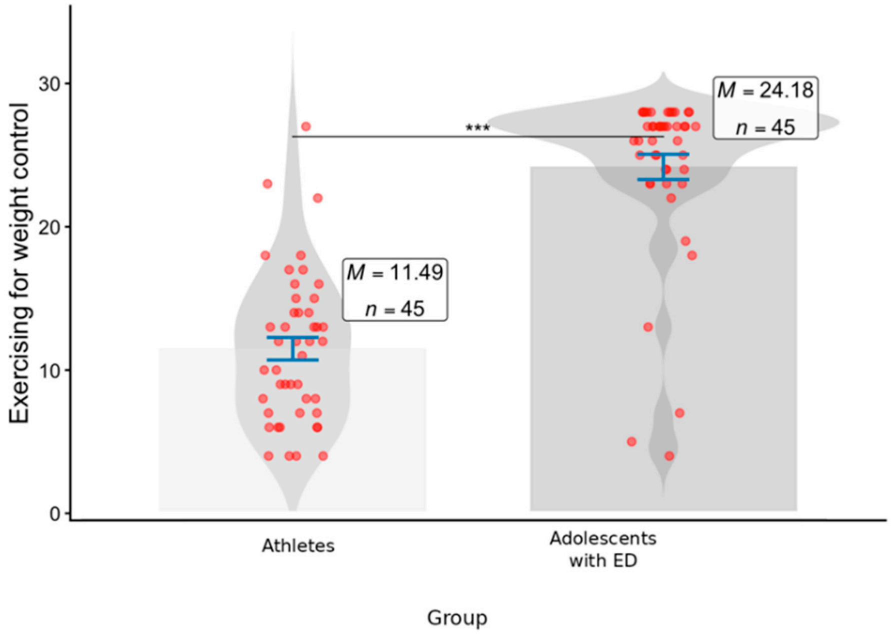

Figure S20. Differences between groups in terms of the level of the variable Exercising for weight control.

Note: * . $p < .05$, ** . $p < .01$, *** . $p < .001$, *ni - insignificant result.* Blue error whiskers indicate the standard error of the mean (SE). Red dots indicate the values of individual observations.

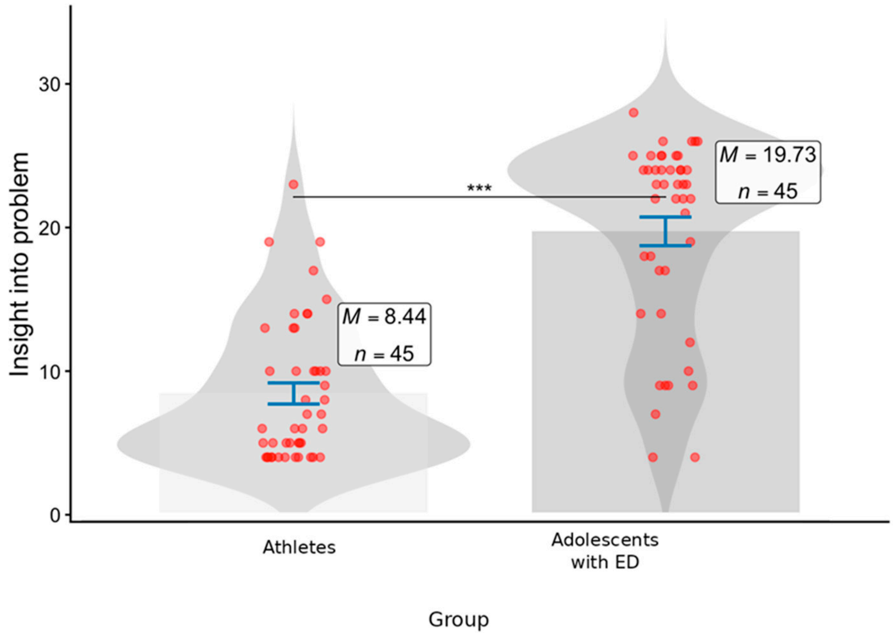

Figure S21. Differences between groups in terms of the level of the variable Insight into problem.

Note: * . $p < .05$, ** . $p < .01$, *** . $p < .001$, *ni - insignificant result.* Blue error whiskers indicate the standard error of the mean (SE). Red dots indicate the values of individual observations.

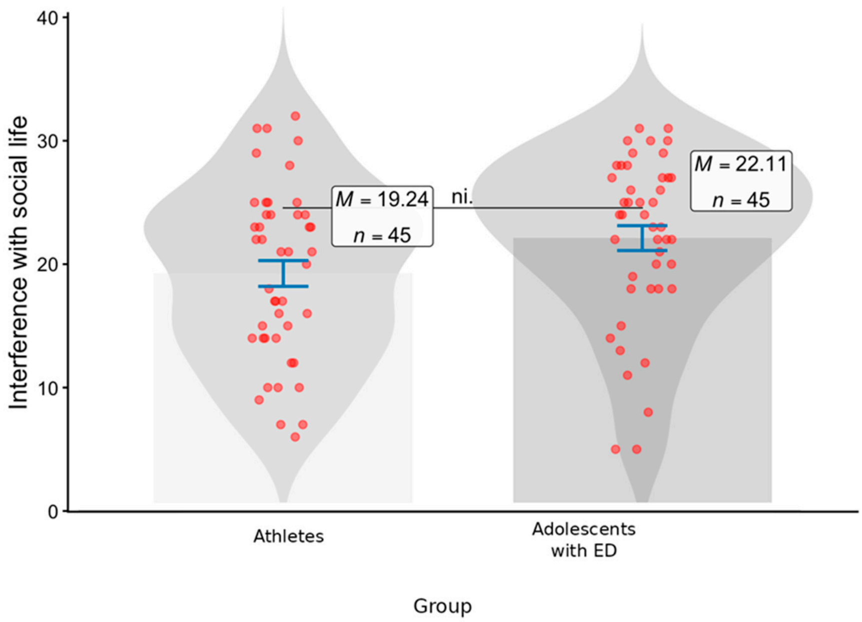

Figure S22. Differences between groups in terms of the level of the variable Interference with social life.

Note: * . $p < .05$, ** . $p < .01$, *** . $p < .001$, *ni - insignificant result.* Blue error whiskers indicate the standard error of the mean (SE). Red dots indicate the values of individual observations.

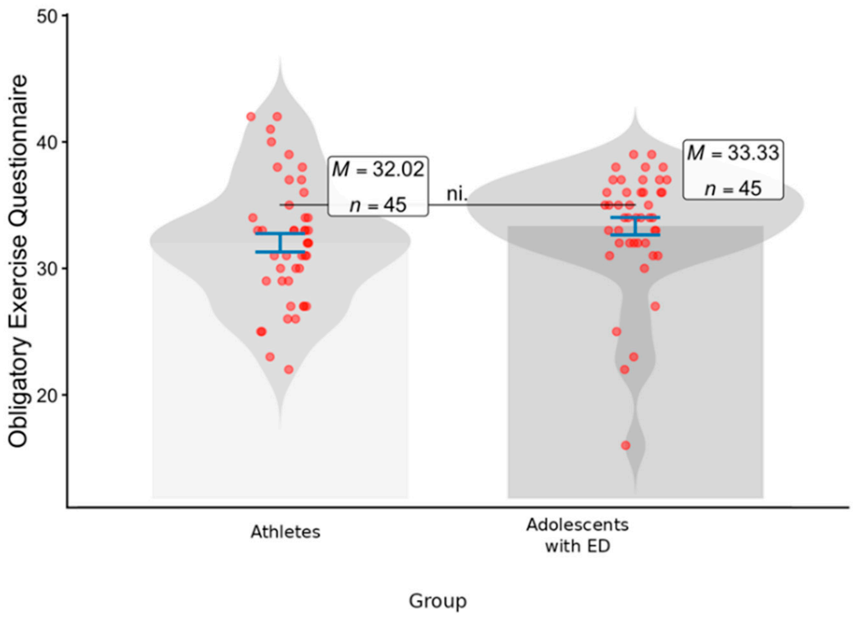

Figure S23. Differences between groups in terms of the level of the variable Obligatory Exercise Questionnaire.

Note: * . $p < .05$, ** . $p < .01$, *** . $p < .001$, *ni - insignificant result.* Blue error whiskers indicate the standard error of the mean (SE). Red dots indicate the values of individual observations.

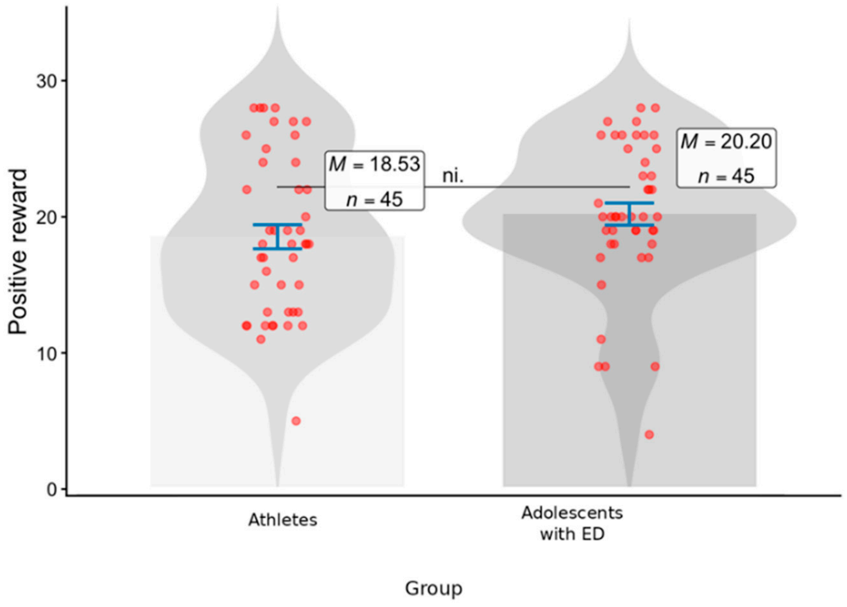

Figure S24. Differences between groups in terms of the level of the variable Positive reward.

Note: * . $p < .05$, ** . $p < .01$, *** . $p < .001$, *ni - insignificant result.* Blue error whiskers indicate the standard error of the mean (SE). Red dots indicate the values of individual observations.

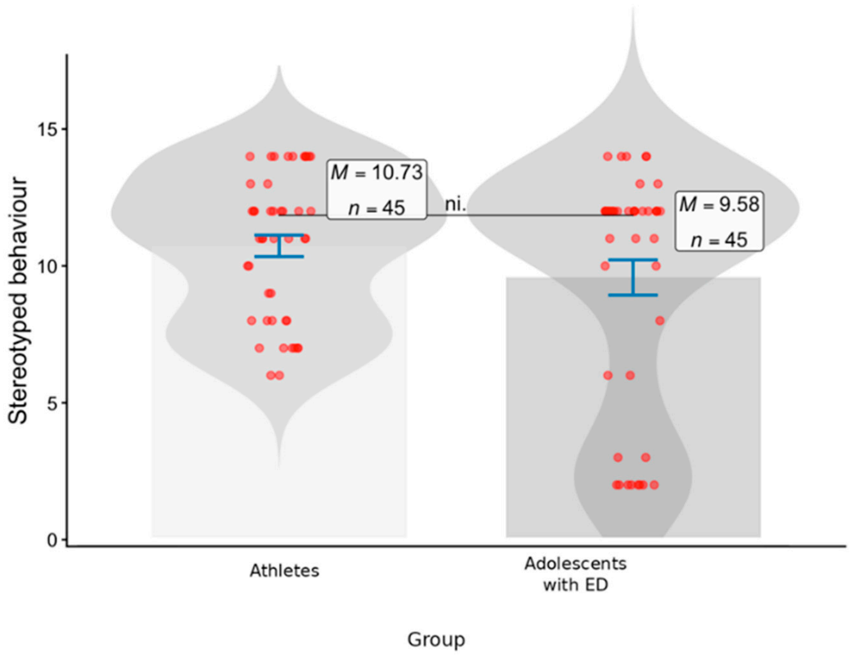

Figure S25. Differences between groups in terms of the level of the variable Stereotyped behaviour.

Note: * . $p < .05$, ** . $p < .01$, *** . $p < .001$, *ni - insignificant result.* Blue error whiskers indicate the standard error of the mean (SE). Red dots indicate the values of individual observations.

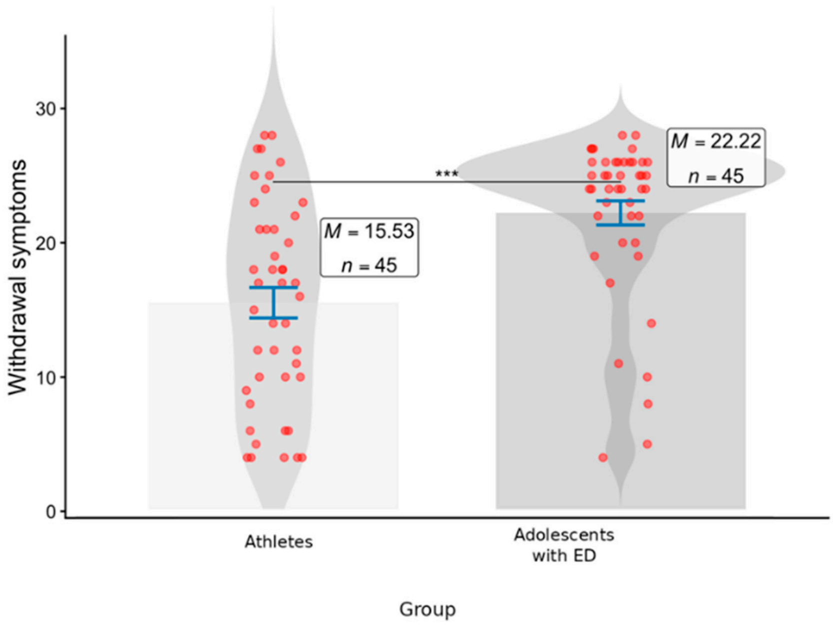

Figure S26. Differences between groups in terms of the level of the variable Withdrawal symptoms.

Note: * . $p < .05$, ** . $p < .01$, *** . $p < .001$, *ni - insignificant result.* Blue error whiskers indicate the standard error of the mean (SE). Red dots indicate the values of individual observations.
